# Mevalonate pathway-triggered phase transition of injectable hydrogel for cholesterol-downregulated therapy of osteoarthritis

**DOI:** 10.1016/j.bioactmat.2025.06.047

**Published:** 2025-06-28

**Authors:** Akhmad Irhas Robby, Ee Hyun Kim, Kang Moo Huh, Eun-Jung Jin, Ki Dong Park, Sung Young Park

**Affiliations:** a Chemical Industry Institute, Korea National University of Transportation, Chungju, 27469, Republic of Korea; b Department of Chemical & Biological Engineering, Korea National University of Transportation, Chungju, 27469, Republic of Korea; c Department of Biomedical Materials Science, Graduate School of JABA, Wonkwang University, Iksan, Jeonbuk State, 54538, Republic of Korea; d Department of Polymer Science and Engineering, Chungnam National University, Daejeon, 305-764, Republic of Korea; e Department of Molecular Science and Technology, Ajou University, Woncheon, Yeongtong, Suwon, 443-749, Republic of Korea

**Keywords:** *Osteoarthritis*, *Injectable hydrogel*, *Coenzyme A*, *Mevalonate pathway*, *Theragnostic*

## Abstract

Dysregulation of mevalonate pathway, an essential metabolic route involving coenzyme A (CoASH) and cholesterol, contributes significantly to escalating cartilage degradation. Existing treatments rely on the simvastatin delivery *via* tunable sol-gel transition mechanisms of injectable hydrogel. However, those methods suffer from lack of controllable drug release by selective phase transition under distinct disease microenvironment. Herein, we developed an aberrant lipid metabolism microenvironment-activated phase transition (normal condition: gel-gel, abnormal condition: gel-sol) with targeted drug release for synergistic treatment of osteoarthritis (OA). Naked-eye diagnosis and therapy of OA through cholesterol downregulation using an injectable hydrogel were based on the simvastatin-loaded nanoparticles embedded in hexanoyl glycol chitosan (HGC-SIM@PAA-MnO_2_-cPDA or SIM gel). The interaction between highly expressed CoASH in OA and PAA-MnO_2_ in SIM gel altered the hydrophobic–hydrophilic balance and gelation temperature, triggering the OA-sensitive gel-sol transformation. Naked-eye gel-sol transformation was observed after incubating SIM gel with OA chondrocyte models, including acetyl-CoA-induced wild-type (WT + CoA), *NudT7*^*−/−*^ knockout (N7KO), and *Acot12*^*−/−*^ knockout (A12KO). Because of the simvastatin release after gel-sol transition, OA-related enzymes and genes, including antioxidant enzymes (*Sod2*), cartilage degradation genes (*Adamts4*), and cholesterol synthesis-related enzymes (*Mvk*), were downregulated. *In vivo* studies revealed gel-sol transformation in destabilized medial meniscus of OA mice (DMM WT, N7KO, and A12KO) at 4–8 weeks post-injection, with significantly reduced cartilage degradation, demonstrating theragnostic capability of SIM gel. Thus, SIM gel offers a potential approach for future synergistic OA diagnosis and therapy.

## Highlights

• Controllable Sol-Gel/Gel-Sol phase transition was affected by mevalonate pathway.
• Conductive injectable hydrogel was designed to control drug release with phase transition.
• OA microenvironment-triggered simvastatin release downregulates *Mvk, Sod2* and *MMP13*.
• *In vivo* simvastatin release in DMM OA mice reduced the cartilage degradation.
• Distinct phase transition can be used for naked-eye hydrogel-based OA detection.

## Graphical abstract

**Figure:**
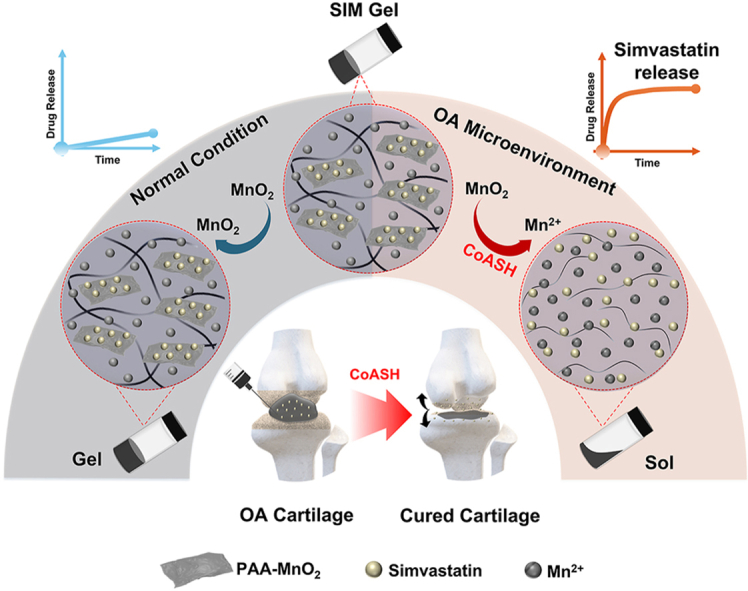

## 1 Introduction

Osteoarthritis (OA) is a degenerative joint dysfunction that involves cartilage damage, structural changes in the underlying bone, and synovial inflammation and is the most prevalent disability, affecting more than 500 million people worldwide [[1], [2], [3]]. The complex pathogenesis of OA involves altered metabolic pathways, including glycolytic metabolism, mitochondria dysfunction, peroxisomal β-oxidation impairment, and abnormal lipid metabolism, which cause certain abnormalities in the cartilage [[4], [5], [6], [7]]. Coenzyme A (CoASH) and its derivative, acetyl-CoA, play a pivotal role in metabolic processes crucial for energy production and synthesis of essential biomolecules [[8], [9], [10]]. Recent studies have explored its connection with the pathogenesis of OA. Dysregulation of CoASH or acetyl-CoA levels in OA compared to non-OA condition can lead to abnormal lipid metabolism and accumulation of fatty acids in joints [[11], [12], [13]]. This accumulation can increase oxidative stress and inflammation, which are critical factors in the pathogenesis of OA. The mevalonate pathway, a critical metabolic pathway that converts acetyl-CoA to cholesterol, isoprenoids, and other essential lipids, also plays a significant role in the pathogenesis of OA [14,15]. Dysregulation of this pathway can alter the biosynthesis of crucial molecules such as cholesterol and non-sterol isoprenoids, disrupting cell signaling, protein prenylation, and membrane integrity [[16], [17], [18]]. Such alterations are linked to increased inflammation and cartilage degradation, which are common features of OA [19,20]. Statins, like simvastatin, target this pathway, inhibit 3-hydroxy-3-methylglutaryl coenzyme A (HMG-CoA) reductase, the rate-limiting enzyme of the mevalonate pathway, and thus reduce inflammatory responses and prevent further cartilage damage in OA [[21], [22], [23], [24]]. Moreover, in aging-related metabolism, the elevation of CoASH concentration due to *NudT7*^*−/−*^ (N7KO) and *Acot12*^*−/−*^ (A12KO) knockouts in senescent chondrocytes is a unique property of OA [25,26]. Hence, monitoring the expression of CoASH as an OA biomarker in cartilage could be crucial in addressing OA-related issues [27]. However, current detection platforms, such as liquid chromatography and polymerase chain reaction (PCR), are inconvenient for monitoring OA progression because of their complex operating procedures and high maintenance costs [[28], [29], [30]].

Drug therapy with opioids and nonsteroidal anti-inflammatory drugs has been employed as an alternative to surgery for OA treatment [[31], [32], [33]]. These medications effectively relieve joint pain; however, the risk of damage to the kidney, liver, and cardiovascular system limits their long-term use. Among other types of drugs, simvastatin, which is widely used for its cholesterol-lowering properties, influences metabolic pathways involving acetyl-CoA and has emerged as a potential therapeutic approach [34,35]. By modulating this pathway, simvastatin can potentially reduce the metabolic disturbances associated with OA, thereby alleviating joint inflammation and slowing cartilage degradation [14,16,34]. Given its effect on the mevalonate pathway, the role of simvastatin extends beyond cholesterol reduction, offering potential benefits for managing OA-related metabolic imbalances [22,23]. Incorporating simvastatin into a temperature-sensitive injectable hydrogel ensures the drug is administered directly to the lesion site. Temperature-sensitive injectable hydrogels, a type of hydrogel that possesses sol-to-gel transformation at body temperature (37 °C) owing to the change in hydrophobic-hydrophilic balance, are widely used for drug delivery systems owing to their multiple advantages, such as biocompatibility, facile administration and facilitating local treatment [[36], [37], [38]]. Various natural and synthetic polymers, including chitosan and its derivatives (hexanoyl glycol chitosan), methyl cellulose and Pluronic, become common options for constructing injectable hydrogel-based drug delivery systems [[39], [40], [41]]. However, controlling the release of the drug from injectable hydrogel can be challenging due to its retention in the hydrogel matrix [[42], [43], [44]]. Thus, modifying the structure and properties of injectable hydrogel to control the drug release selectively is necessary; for example, manipulating the interaction between injectable hydrogel and an OA biomarker (such as CoASH), which alters the physical form and properties of the gel to allow on-demand release of simvastatin.

Herein, we designed a novel lipid imbalance-induced gel-sol phase change with triggered drug release from simvastatin-loaded temperature-sensitive injectable conductive hydrogel (HGC-SIM@PAA-MnO_2_-cPDA or SIM gel) for OA theragnosis. A unique gel-sol transition was achieved under exposure of highly expressed CoASH due to the change of hydrophobic–hydrophilic balance and shift in the gelation temperature. The naked-eye gel-sol transition could be observed in OA chondrocytes (N7KO and A12KO), and simvastatin can be selectively released in OA cartilage for repairing the damaged cartilage as indicated by the downregulation of OA-related enzymes and genes. By employing this approach, a novel theragnostic system for a synergistic controlled release of simvastatin in OA cartilage and a naked-eye OA monitoring based on the CoASH level can be achieved, demonstrating a promising potential for joint dysfunction theragnostic.

## 2 Experimental section

### 2.1 Materials

Glycol chitosan, hexanoic anhydride, polyacrylic acid (PAA, Mw: 250000 g/mol), dopamine hydrochloride, potassium permanganate (KMnO_4_), 2-(*N*-morpholino) ethane sulfonic acid (MES), coenzyme A, simvastatin, calcium chloride (CaCl_2_), and sodium hydrogen phosphate (Na_2_HPO_4_), were purchased from Sigma-Aldrich (Yongin-si, Gyeonggi-do, Korea). Phosphate-buffered saline (PBS; pH 7.4) was obtained from Bioneer Corp. (Daejeon, Korea). Carbonized polydopamine (cPDA) was obtained *via* hydrothermal carbonization of polydopamine in Teflon-lined reactor at 180^o^C for 8 h [45]. Temperature-sensitive hexanoyl glycol chitosan (HGC) was synthesized based on the previous report by reacting glycol chitosan with hexanoic anhydride [46]. Penicillin-streptomycin, minimum essential medium (MEM), Roswell Park Memorial Institute (RPMI)−1640 medium, and Trypsin-ethylenediaminetetraacetic acid (0.05 % w/v trypsin-EDTA 1X), were bought from Gibco BRL (NY, USA). Fetal bovine serum (FBS) was purchased from Merck (Darmstadt, Germany). Calcein AM and propidium iodide (PI) were obtained from Life Technologies (Carlsbad, CA, USA).

### 2.2 Characterizations

^1^H-NMR spectra were obtained using a Bruker AVANCE 400 MHz spectrometer (Waltham, USA). UV–vis spectra were measured using Optizen 2120UV spetrophotometer, Mecasys, South Korea. Dynamic light scattering (DLS) profiles were obtained using Zetasizer Nano, Malvern, Kassel, Germany. Scanning electron microscopy (SEM) images was captured with a JSM-6700F, JEOL, Japan. Confocal images were observed *via* the ECLIPSE Ti2-E confocal microscope, Nikon, Japan. The rheological properties were evaluated by a HAAKE MARS modular advanced rheometer (20 mm plate). Compression and adhesive tests were performed using a universal testing machine, SurTA 1A, Chemilab Co., South Korea. The electroconductivity measurements of samples were conducted using electrochemical impedance spectrometer (EIS, CS350, CorrTest Instrument, China), sourcemeter (Keithley 2450, Tektronik, USA) and a wireless device (Arduino Uno ATmega328 P Processor micro-controller and AppGosu Bluetooth module).

### 2.3 Synthesis of simvastatin drug-loaded mineralized nanoparticle with polyacrylic acid-manganese oxide (SIM@PAA-MnO_2_)

The polyacrylic acid-manganese oxide (PAA-MnO_2_) was synthesized by mixing PAA solution (10 %) with KMnO_4_ (75 mg) and MES solution (pH 6, 0.1 M) for 4 h at room temperature. The solution was then dialyzed (MWCO: 3.5 kDa) against DDW and freeze-dried [47]. For loading simvastatin into the PAA-MnO_2_, 5 mg of simvastatin (in 0.5 mL DMSO) and 50 mg of PAA-MnO_2_ (in 4.5 mL PBS pH 7.4) were mixed and stirred for 24 h at room temperature, followed by dialysis against DDW (MWCO:1 kDa) and freeze-drying process.

### 2.4 Synthesis of SIM@PAA-MnO_2_ and carbonized polydopamine (cPDA)-loaded HGC hydrogel (SIM gel)

SIM gel was fabricated by incorporating SIM@PAA-MnO_2_ and cPDA (to enhance adhesive property) into the HGC. In brief, HGC solution (40 mg/mL in PBS pH 7.4) was mixed with mineralized solution of SIM@PAA-MnO_2_ (containing 1 mg of SIM@PAA-MnO_2_, CaCl_2_ 2 M and Na_2_HPO_4_ 2 M) and cPDA (2 mg) and swelled in refrigerator (4 °C) until become homogenous. The mixture will form a sol at room temperature (25 °C), while it becomes a gel at physiological temperature (37 °C), named as SIM gel. For the control, the mixture of all compound excluding simvastatin was used and named as PD gel (without simvastatin).

### 2.5 Preparation of CoASH-triggered gel-sol formation of SIM gel

The as-prepared SIM pre-gel solution (1 mL) was incubated at 37 °C until it became gel. Subsequently, 100 μL of CoASH solution (10 mM) was added, and allowed to react depending on time (30, 60, 90 and 120 min) at 37 °C. The change in gel-sol phase was observed visually (naked eye) and can be quantitatively measured using rheometer by determining the gelling temperature between before and after CoASH treatments (temperature sweep, 20–50^o^C, frequency (f) = 1 Hz, strain (γ) = 0.1, P20mm/Ti plate rotor).

### 2.6 *In vitro* experiments

Murine primary articular chondrocytes were isolated from wildtype (WT), *Acot12*^*−/−*^ knockout (A12KO), and *Nudt7*^*−/−*^ knockout (N7KO) postnatal mice. In brief, dissected articular cartilage was digested with collagenase D (Roche, 11088858001) and filtered through a 70-mm cell strainer. Primary chondrocytes were cultured with low glucose (1 g/L) Dulbecco's modified Eagle's medium (DMEM; Gibco) supplemented with 10 % FBS (Gibco, 12483020) and Penicillin-Streptomycin (Gibco, 15140122) in a 37 °C incubator with 5 % CO_2_. The WT, N7KO, A12KO and WT + 50 mM of acetyl-CoA (CoA) were treated for 24 h with HGC, PD/gel (without simvastatin), or SIM gel. The gel sol transformation of each sample was observed via naked eye and rheological analysis.

### 2.7 Live and dead assay

Immortalized mouse articular chondrocytes (iMAC) were incubated with gels for 24 h. Live/Dead staining was performed following the manufacturer's instruction (Molecular probes, #MP03224). Images were captured using EVOS FL Auto Cell Imaging System.

### 2.8 Conductivity measurement and wireless sensing of SIM gel

The electrochemical characterization of SIM gel was conducted using sourcemeter and wireless sensing apparatus (2-electrode DC set-up). SIM gel was connected to the sourcemeter using copper plate, and the change in resistance during gel-sol formation was read by sourcemeter. For wireless sensing, a Bluetooth module (AppGosu), a microcontroller (Arduino Uno) and a smartphone were arranged and connected to the SIM gel. The resistance graph was displayed on the smartphone by activating Bluetooth connection [48,49].

### 2.9 *In vivo* animal model

All animal studies followed the approval from the Wonkwang University Animal Care and Use Committee and the institutional guidelines (WKU23-37). Destabilization of the medial meniscus (DMM) surgery for OA induction was operated on 8-weeks-old male WT, A12KO and N7KO C57BL/6 mice. A total of 20 μL of hydrogel was intra-articularly injected into the knee joint at the time of DMM surgery. Mice were euthanized, and knee joints were harvested 8 weeks post-surgery for analysis. OA progression was assessed post-mortem by histological analysis (Safranin O staining and OARSI scoring). The “Sham group” refers to the contralateral (non-operated) knee and served as a healthy control. The "No gel" group, in which DMM surgery was performed without hydrogel treatment, represented the OA condition.

### 2.10 Immunohistochemistry and histological staining

The knee joints were embedded in paraffin after fixation in 10 % neutral buffered formalin for 24 h and decalcification in 14 % EDTA (pH 7.4) for seven days. 5 μm-sliced sections were rehydrated, counterstained with Weigert's iron hematoxylin solution, stained with fast green solution, then stained in safranin O solution. To assess anabolic responses in cartilage tissue, mouse knee joints were harvested at 8 weeks post-DMM surgery and SIM gel treatment. Paraffin-embedded sections (5 μm) were deparaffinized, rehydrated, and subjected to antigen retrieval in citrate buffer (pH 6.0). Sections were blocked with 5 % bovine serum albumin (BSA) and incubated overnight at 4 °C with primary antibodies against type II collagen (COL2A1, 1:200; Santa Cruz Biotechnology, sc-7764) and aggrecan (Acan, 1:100; Novus Biologicals, NB100-74350). For cholesterol BODIPY staining, PBS-washed sections were incubated with 50 mM BODIPY-cholesterol (Cayman Chemical, 24618) for 1 h, and counterstained with DAPI (4′,6-diamidino-2-phenylindole dihydrochloride; Molecular Probe D1306). For biocompatibility validation, the skin, kidney, liver, and spleen were harvested at the time of sacrifice, fixed in 10 % formalin, embedded in paraffin, and sectioned at 5 μm thickness. Hematoxylin and eosin (H&E) staining and immunohistochemistry were performed using antibodies against F4/80 (1:200; Cell Signaling Technology, #9664) and cleaved caspase-3 (1:200; Abcam, #ab111101).

### 2.11 *In silico* data analysis

Human chondrocytes data (GSE241126, GSE180467, and GSE29868) were collected from Gene Expression Omnibus (GEO) repository, differentially expressed genes with *p* < 0.05 simultaneously in both databases were selected and analyzed using The Database for Annotation, Visualization and Integrated Discovery (DAVID).

## 3 Result and discussion

### 3.1 Design and mechanism of CoASH-responsive injectable and conductive SIM gel with controlled drug release

**Fig. 1:**
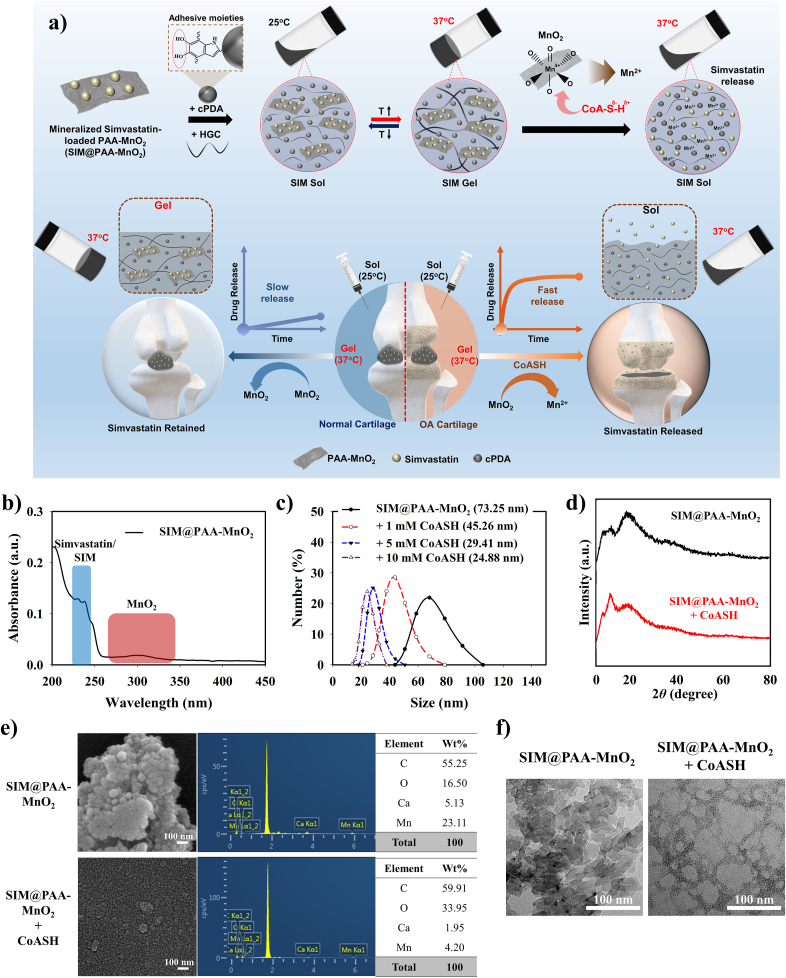
**Design of SIM@PAA-MnO_2_ nanoparticles and the effect of CoASH on its morphology. a)** Schematic illustration of CoASH-induced gel-sol transformation of SIM gel for OA theragnostic. **b)** UV–vis spectra of SIM@PAA-MnO_2_ nanoparticles. **c)** DLS measurement, **d)** XRD patterns, **e)** SEM-EDX profiles, and **f)** TEM images of SIM@PAA-MnO_2_ nanoparticles before and after treatment with CoASH (10 mM).

A thermo-responsive injectable hydrogel (SIM gel) with gel-sol phase transition induced by the high expression of CoASH having a synergistic diagnosis and therapeutic approach for OA at the physiological temperature (37 °C) was constructed. This hydrogel was synthesized by combining CoASH-sensitive simvastatin-loaded PAA-MnO_2_ (SIM@PAA-MnO_2_ nanoparticles) with an additional adhesive cPDA nanoparticles (owing to the adhesive catechol moieties) into an injectable thermo-responsive hexanoyl glycol chitosan (HGC). At room temperature (25 °C) or lower, the sol phase (SIM pre-gel) was obtained, which was transformed to the gel phase (SIM gel) when the temperature increased to 37 °C. As CoASH was incorporated into SIM gel, a gel-to-sol transition occurred due to the reduction of MnO_2_ (Mn^4+^) to Mn^2+^ by CoASH and the resulting change in the hydrophobicity of SIM gel. Hence, this gel-sol transformation at physiological temperature can be employed to design a unique system for monitoring the level of CoASH in OA cartilage *via* naked-eye, as well as for OA therapy because of the release of simvastatin in the OA microenvironment (Fig. 1a).

The structure of the synthesized thermoresponsive HGC was evaluated using H-NMR (Fig. S1). The degree of substitution (DS) of the hexanoyl group was estimated to be 0.55 based on the ratio between the glucopyranosyl ring and the hexanoyl group. The presence of hydrophobic hexanoyl group in HGC facilitates temperature-induced enhanced hydrophobic interactions that become the driving force for thermosensitive gelling behavior [40,46]. On the other hand, the presence of PAA-MnO_2_ as a drug carrier for simvastatin plays a crucial role on OA microenvironment responsiveness, as redox-responsive MnO_2_ will interact with abundant CoASH in OA microenvironment and will change the hydrophobic-hydrophilic balance in the as-synthesized SIM gel [26,47]. As shown in Fig. S2a, XRD profile of PAA-MnO_2_ showed a peak at *2θ* of 19.5° corresponding to the MnO_2_, which was diminished after treatment with CoASH. The degradation of PAA-MnO_2_ by CoASH was also observed from SEM-EDX analysis (Fig. S2b). The change in nanoparticle morphology along with the decrease of elemental Mn composition before (23.82 %) and after (4.64 %) CoASH treatment revealed the cleavage of MnO_2_ affected by redox reaction with CoASH. Moreover, TEM images confirmed the breakage of MnO_2_ after CoASH treatment, indicated by the change in the particle morphology compared to the nanoparticle before CoASH treatment (Fig. S2c). After loading the simvastatin into the PAA-MnO_2_, the as-prepared SIM@PAA-MnO_2_ nanoparticles were then characterized using UV–vis spectrometry. The absorbance peaks at 240 and 300 nm correspond to simvastatin and MnO_2_, revealing the successful loading of simvastatin onto PAA-MnO_2_ (Fig. 1b) [47,50]. The CoASH responsiveness of SIM@PAA-MnO_2_ nanoparticles was assessed by observing the changes in the particle size before and after treatment with various concentrations of CoASH (1, 5, or 10 mM). As shown in Fig. 1c, the particle size of SIM@PAA-MnO_2_ (73.25 nm) was reduced after being exposed to CoASH; the reduction was greater at higher concentrations (1 mM = 45.26 nm, 5 mM = 29.41 nm, 10 mM = 24.88 nm). This smaller particle size was due to the CoASH-induced breakdown of MnO_2_ into Mn^2+^, with a simultaneous release of simvastatin from SIM@PAA-MnO_2_. This phenomenon was further confirmed by XRD and SEM-EDX analyses as SIM@PAA-MnO_2_ nanoparticles showed a diminishing of MnO_2_ diffraction peak at *2θ* of 19.2° along with morphological-elemental composition changes between before (% Mn = 23.11 %) and after (% Mn = 4.20 %) CoASH treatment (Fig. 1d–e). TEM images of SIM@PAA-MnO_2_ nanoparticles also indicated the change in the particle morphology after the SIM@PAA-MnO_2_ nanoparticles was exposed to the CoASH, confirming the nanoparticles responsiveness towards CoASH (Fig. 1f). The release profile of simvastatin revealed CoASH and PAA-MnO_2_ interaction as the main inducing factor for simvastatin release, with facilitated release at higher CoASH concentrations compared with at lower concentrations or in the absence of CoASH (Fig. S3). This distinct rate of simvastatin release closely correlated with the degradation of MnO_2_ (as a drug carrier) to Mn^2+^ affected by different CoASH level that is presence in OA microenvironment. Moreover, simvastatin-incorporated HGC gel (SIM@HGC) showed burst release of simvastatin even without CoASH treatment owing to the absence of PAA-MnO_2_ that retained the simvastatin, revealing the inability of only HGC to control on-demand release of simvastatin in OA microenvironment. These data confirms that the presence of PAA-MnO_2_ for simvastatin carrier can control the sustain release depend on concentration of CoASH.

### 3.2 CoASH-sensitive gel-sol phase transition of thermo-responsive SIM gel

**Fig. 2:**
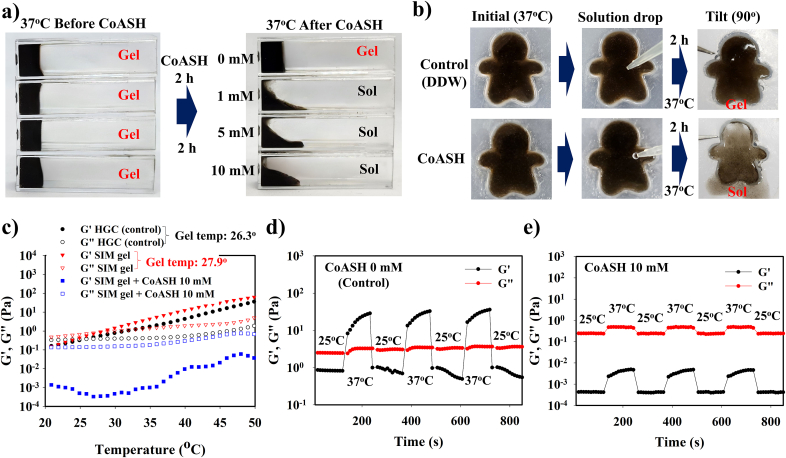
**Gel-sol transition of SIM gel triggered by CoASH. a)** Gel-sol transformation of SIM gel at various CoASH concentrations (0–10 mM), and **b)** different gel-sol transition between shape-molded SIM gel treated with DDW and 10 mM CoASH after 2-h incubation at 37 °C. **c)** Rheological temperature sweep of SIM gel without and with 10 mM CoASH treatment for 2 h. Rheological reversibility test of SIM gel at 25° and 37 °C **d)** without and **e)** with 10 mM CoASH treatment.

SIM@PAA-MnO_2_ and PDA nanoparticles were incorporated into HGC to obtain a CoASH-sensitive SIM gel at body temperature (37 °C). SIM gel exhibited a maximum swelling ratio of approximately 111.53 % at 37 °C (Fig. S4). CoASH-induced gel-sol transformation of SIM gel was determined by treating SIM gel with various concentrations of CoASH for different time durations. As shown in Fig. 2a and S5, the gel form was observed in SIM gel at the initial state of 37 °C. As the reaction time was longer, the sol was formed in SIM gel treated with CoASH (1, 5, or 10 mM) at 37 °C; particularly, the completed sol formation was achieved by treating with 10 mM CoASH for 120 min compared with 1 and 5 mM CoASH for 120 min (which were more viscous sols than that using 10 mM). In contrast, SIM gel without CoASH treatment (treated only with DDW) remained stable even after 120 min, which demonstrated the effect of CoASH on inducing the gel-sol phase transition. A clear visualization of gel-sol transformation in SIM gel can be observed in the molded SIM gel after treatment with DDW and CoASH for 2 h at 37 °C (Fig. 2b). DDW-treated SIM gel retained its shape even when tilted at 90°. Otherwise, the shape of the molded SIM gel disintegrated after exposure to 10 mM CoASH as the formed sol dropped after tilting. These gel-sol transformations were further quantitatively analyzed by determining the gelation temperature using temperature-sweep rheological analysis (Fig. 2c and S6a). Before incorporation of SIM@PAA-MnO_2_ nanoparticles (HGC gel only), the gelling temperature was 26.3 °C, whereas after incorporation (SIM gel), the gelling temperature was increased to 27.9 °C, which confirmed that HGC and SIM gel will form a gel at body temperature. In the presence of CoASH, the loss modulus (G”) was continuously higher than the storage modulus (G’), and the gelation temperature was not achieved even up to 50 °C, indicating that sol formation occurred only after CoASH treatment. Moreover, a reversibility test towards alternating temperature (25° and 37 °C) demonstrated reversible sol-gel-sol transition of SIM gel in the absence of CoASH (Fig. 2d), whereas irreversible sol-gel-sol characteristics were observed in the presence of CoASH (Fig. 2e–S6b-c). These results revealed the effect of CoASH on the gel-sol behavior of SIM gel, which involved breakdown of MnO_2_ into Mn^2+^, affecting the hydrophobic–hydrophilic balance and shifting the gelation temperature of SIM gel. The conversion of MnO_2_ to Mn^2+^ in SIM gel can be confirmed by zeta potential measurement. As the CoASH concentration increased from 1 to 100 mM, the zeta potential became more positive compared to that without CoASH treatment (−18.5 mV), particularly after 120 min of incubation (1 mM = −10.8 mV, 5 mM = −7.5 mV, 10 mM = −6.8 mV, 50 mV = −6.7 mV, and 100 mM = −6.4 mV) (Fig. S7a). The change in zeta potential after CoASH treatment clearly indicates the occurrence of redox mechanism between MnO_2_ and CoASH as a reducing agent, degrading MnO_2_ into Mn^2+^ which causes more positive zeta potential. Dynamic light scattering (DLS) measurement of SIM gel further confirmed that the hydrophobic–hydrophilic balance was affected by CoASH. Before CoASH treatment, the bigger particle size was found to be approximately 178.1 nm at 37 °C, owing to the gel state formation triggered by hydrophobic interaction-induced aggregation. When different concentration of CoASH was introduced, the particle size was reduced to around 81.5–100.8 nm as the hydrophobicity was disrupted, causing less aggregation and gel-sol transformation at 37 °C. Otherwise, SIM gel without CoASH treatment showed negligible change in particle size and form at 37 °C (177.3 nm, gel), revealing the mechanism of CoASH-induced alteration in the hydrophobic–hydrophilic balance of SIM gel (Fig. S7b).

**Fig. 3:**
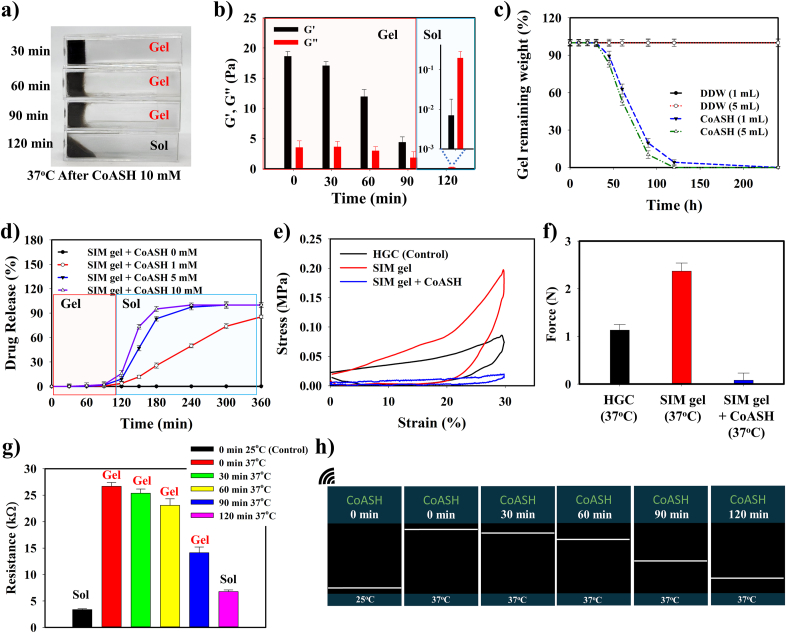
**Change of physical and electrochemical properties of SIM gel in the presence of CoASH. a)** Gel-sol transformation of SIM gel treated with 10 mM CoASH at 37 °C depends on the incubation time. **b)** G′ and G” of CoASH-treated SIM gel depend on the incubation time. **c)** SIM gel remaining weight after treatment with low and excess DDW and CoASH. **d)** Simvastatin release profile from SIM gel after treating with different concentrations of CoASH. **e)** Compression test and **f)** adhesive test of SIM gel without and with 10 mM CoASH treatment. **g)** Source meter measurement and **h)** wireless sensing display (resistance graph) of 10 mM CoASH-treated SIM gel depending on the incubation time.

The gel-to-sol phase transformation of SIM gel facilitated the release of simvastatin to treat OA after the sol is formed. In the presence of 10 mM CoASH, the minimum treatment duration required to achieve gel-sol transformation was 120 min (Fig. 3a). A gradual alteration in the G′ and G” ratio at 37 °C was observed as CoASH treatment time was increased from 30 to 120 min. The gel form was retained from 0 to 90 min, whereas the overlapping of G” over G’ at 120 min indicated sol formation (Fig. 3b). This gel-sol transition at 37 °C can be evaluated by measuring the remaining gel weight between SIM gel exposed to CoASH and DDW in small (1 mL) and excess amounts (5 mL). DDW-treated SIM gel did not show weight reduction in contrast to CoASH-treated SIM gel, which reduced dramatically owing to the phase change to sol, revealing that SIM gel was stable in DDW; otherwise, the physical characteristics of SIM gel were affected by CoASH (Fig. 3c). Furthermore, the controlled simvastatin release behavior was determined during the gel-sol transition under various CoASH concentrations. In the gel phase below 120 min-reaction, the release of simvastatin was inhibited (maximum release of 15.8 % at 10 mM CoASH); however, simvastatin was completely released into the sol phase after treatment with 10 mM CoASH for 4 h (Fig. 3d). This controlled release behavior confirmed that gel-to-sol transition facilitated the release of simvastatin, which is important for selective drug delivery at the site of OA lesions. The mechanical properties of SIM gel were evaluated using a loading-unloading compression test at 37 °C. Compared with HGC gel, the mechanical strength of SIM gel was superior owing to the hydrogen bonds formed by cPDA contained in SIM gel. After adding CoASH, the mechanical strength of SIM gel weakened as the sol phase was formed (Fig. 3e). The difference in adhesive properties between HGC and SIM gels owing to the presence of cPDA was observed through an adhesive test using UTM. At 37 °C, the adhesive force for SIM gel (2.37 N) was higher than that of HGC gel (1.13 N), whereas CoASH-treated SIM gel possessed the lowest adhesive force (0.08 N) due to sol transformation (Fig. 3f and S8a). These data show the effect of cPDA in SIM gel, in which improved mechanical and adhesive properties would be beneficial for practical applications during injection into OA cartilage. Additionally, the electrochemical properties of SIM gel changed during treatment with CoASH depending on the reaction time and concentration. The source meter data illustrated a gradual decrease in the resistance of SIM gel in the gel form from 0 to 90 min, with lower resistance being achieved when a sol form was used (Fig. 3g). In the concentration-dependent experiment, the resistance of SIM gel decreased significantly when the sol form was obtained after treatment with 1, 5, or 10 mM of CoASH (Fig. S8b). This decrease in resistance of the gel form was related to the gradual gel-to-sol transition in SIM gel, which allowed more ionic or electron mobility and increased the conductivity of the sample, particularly in the sol form. This electroconductivity change can be displayed on a smartphone *via* a wireless sensing device connection, and the resistance graph showed a similar trend to the source meter results, confirming different electroconductivities of SIM gel after CoASH treatment (Fig. 3h and S8c).

### 3.3 *In vitro* study of lipid metabolism disorder-induced gel-sol phase transition of SIM gel in wild-type (WT), N7KO, and A12KO primary articular chondrocytes

**Fig. 4:**
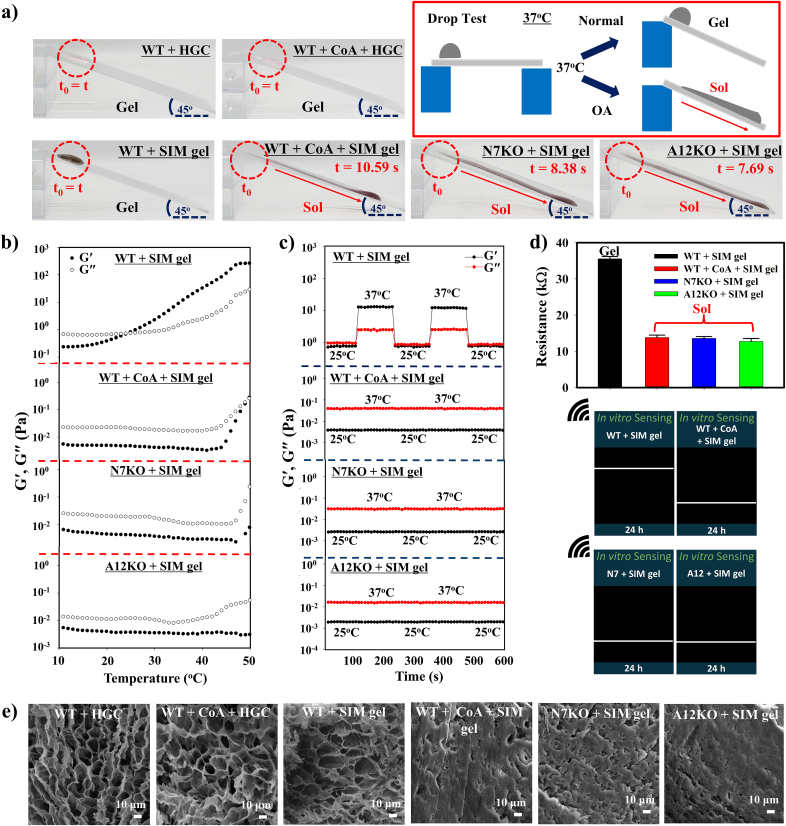
***In vitro* study of SIM gel in the simulated OA microenvironment. a)***In vitro* gel-sol transformation of SIM gel using a simple drop-flowing test, **b)** rheological temperature sweep, **c)** rheological reversibility test, **d)** source meter-wireless sensing measurements, and **e)** SEM imaging of SIM gel incubated with OA chondrocyte models (WT, WT + CoA, N7KO, and A12KO) for 24 h at 37 °C.

The gel-to-sol transformation of SIM gel was determined by its interaction with primary articular chondrocytes (WT, N7KO, and A12KO), which have been reported to express accumulated levels of CoASH in OA chondrocytes [11,12]. Genetic deletion of Nudt7 and Acot12 was confirmed by PCR-based genotyping using allele-specific primers, which yielded distinct wild-type and knockout bands in N7KO and A12KO mice (Fig. S9a). For *in vitro* experiments, three different types of gels, HGC gel (without PD and simvastatin), PD gel (without simvastatin), and SIM gel, were used to compare the gel-sol characteristics and other physical properties of WT, N7KO, and A12KO chondrocytes. The cytotoxicity of these gels towards articular chondrocytes was evaluated *via* live and dead staining assays (green/live: Calcein AM, red/dead: Ethidium homodimer-1) using a confocal microscope. SIM gel and other control gels (HGC and PD gels) showed low cytotoxicity towards articular chondrocytes, indicating the biocompatibility of the gel matrix and incorporated nanoparticles in SIM gels, which can be used for further *in vitro* studies (Fig. S9b). WT, N7KO, or A12KO chondrocytes were subsequently treated with SIM and HGC gels (control) for 24 h at 37 °C to study the changes in the physical properties of the gels. Acetyl-CoA (CoA) (50 mM) was added to WT to validate the role of accumulated CoASH expression in triggering the gel-sol transformation. As shown in Fig. 4a, a simple flow test using an inclined plane (length: 7 cm, angle: 45°) demonstrated distinct forms of HGC and SIM gels after incubation with the OA chondrocyte models. The flow test indicated that the post-treatment SIM gels with WT + CoA (t = 10.59 s), N7KO (t = 8.38 s), and A12KO (t = 7.69) possessed a sol form. In contrast, WT-treated SIM and HGC gels (with WT and WT + CoA) retained their gel integrity and remained at the initial position (t_0_ = t). These phenomena occurred because of the high levels of CoASH expressed in N7KO, A12KO, and CoA-induced WT; the CoASH expression level in WT alone was not sufficient to cleave MnO_2_ in SIM gel, resulting in retaining the gel form. Comparison with HGC revealed that the presence of PAA-MnO_2_-cPDA nanoparticles in SIM gel played a vital role in controlling the gel-sol transformation induced by CoASH in OA chondrocytes. Distinct gel and sol forms in SIM gel were observed using an injection syringe test (Fig. S10). WT-treated SIM gel remained stable in the gel form when injected into water at 37 °C, whereas WT + CoA, N7KO, and A12KO-treated SIM gels were easily dissolved in water owing to their sol form.

The gelation temperature of each SIM gel group was measured by rheological temperature sweep analysis. The gelation temperatures of WT + CoA, N7KO, and A12KO-treated SIM gels were not detected up to 50 °C, with G” consistently higher than G’, indicating their sol form. Otherwise, the gelation temperature of WT-treated SIM gel was observed to be 25.2 °C, which was almost similar to WT and WT + CoA-treated HGC (23.3° and 23.9 °C), confirming the gel formation of those gels at physiological temperature (37 °C) (Fig. 4b and S11a-b). WT-treated SIM gel and HGC gel groups possessed a sol-gel-sol reversibility in 25–37 °C cycles, which was contrary to the irreversible sol form of WT + CoA, N7KO, and A12KO-treated SIM gels (Fig. 4c and S11c-d). The changes in electrochemical properties of SIM gels measured using a source meter and wireless sensing device indicated the sol formation in WT + CoA, N7KO, and A12KO-treated SIM gels (approximately 13 kΩ) and the retained gel form in WT-treated SIM gel, WT-treated HGC gel, and WT + CoA-treated HGC gel (approximately 35–39 kΩ) (Fig. 4d and S12a-b). Moreover, distinct morphologies of SIM gels after incubation with each OA chondrocyte model were evaluated using a scanning electron microscope. The images illustrated interconnected porous networks in WT-treated SIM gel, similar to WT and WT + CoA-treated HGC. In contrast, the collapsed structure indicated the formation of a sol in WT + CoA, N7KO, and A12KO-treated SIM gels (Fig. 4e).

**Fig. 5:**
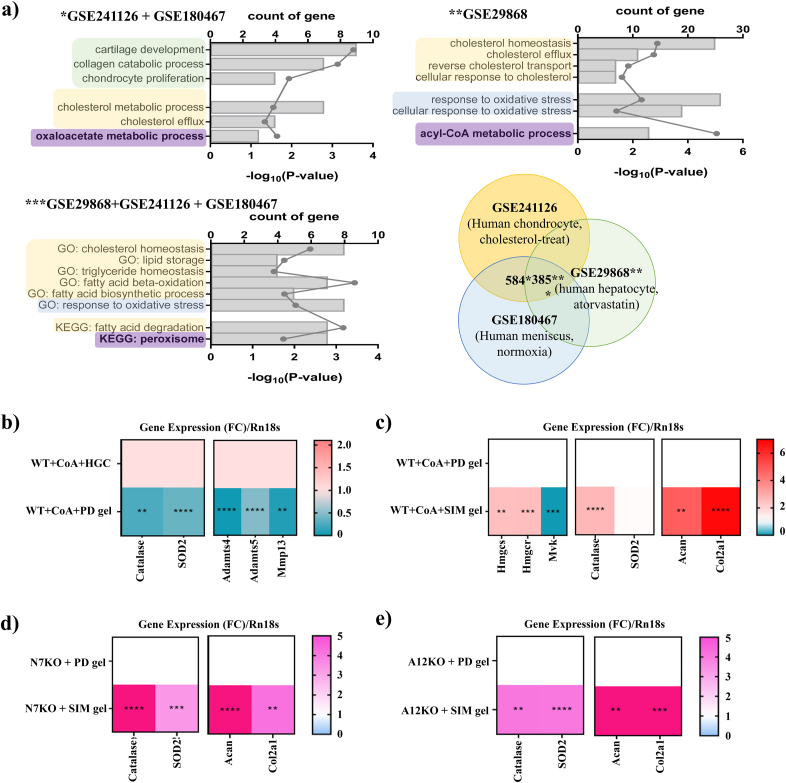
***In vitro* gene expression analysis post-treatment with SIM gel. a)***In silico* analysis of human chondrocyte data (GSE241126, GSE180467, and GSE29868) collected from the Gene Expression Omnibus (GEO) repository. **b)** WT + CoA + HGC and WT + CoA + PD gel. *In vitro* gene expression of anabolic and catabolic factors in OA chondrocytes after treatment with PD gel and SIM gel in the presence of **c)** WT + CoA, **d)** N7KO, and **e)** A12KO. Experiments were performed at least three experiments and data were presented as the means ± SD. Student's t-test and one- or two-way analysis of variance (ANOVA) were used, and significance was defined as ∗ P < 0.05, ∗∗P < 0.01, ∗∗∗P < 0.001, and ∗∗∗∗P < 0.0001.

CoASH and its derivative acetyl-CoA play pivotal roles in metabolic processes closely related to the pathogenesis of OA. Consistent with previous reports, the incorporation of CoA into chondrocytes resulted in a dramatic decrease in anabolic genes, such as aggrecan (*Acan*), collagen type II (*Col2a1*), and cartilage oligomeric matrix protein (*COMP*)*,* and an increase in catabolic genes, such as A disintegrin and metalloproteinase with thrombospondin motifs *4 and 5* (*Adamts4 and Adamts5*) and matrix metallopeptidase 13 (*MMP-13*) (Fig. S13a). In addition, during the delivery of acetyl-CoA into the cartilage of osteoarthritic mice, surgery destabilization of the medial meniscus (DMM) exacerbated the degradation of the cartilage matrix as assessed by safranin O staining and the Osteoarthritis Research Society International (OARSI) scoring (Fig. S13b). Severe cartilage degradation was observed in N7KO and A12KO mice *via* dysregulation of acetyl-CoA metabolism during OA pathogenesis (Fig. S13c) [11,12]. To identify significant biological pathways related to osteoarthritic condition, which is mainly exposed to cholesterol or oxidative stress, 584 genes were collected from the GEO public data of cholesterol-treated human chondrocytes (GSE241126) and human meniscus in normoxia (GSE180467), and gene ontology (GO) pathways were analyzed (Fig. 5a). Changes in collagen catabolic, cholesterol metabolic, and oxaloacetate metabolic processes, which directly or indirectly regulate CoA metabolism, were significantly altered. Furthermore, the enrichment of acyl-CoA metabolic processes in atorvastatin-treated human hepatocytes (GSE29868) and peroxisome enrichment in all three GEO datasets suggest that peroxisomal acyl-CoA regulating protein is a novel factor for cholesterol modification in OA. Introduction of PD gel (without simvastatin) in acetyl-CoA-stimulated primary chondrocytes downregulated the transcriptional levels of antioxidant enzymes, including *catalase* and *Sod2*, as a result of effective scavenging of ROS facilitated by MnO_2_, and consequently downregulated cartilage degradation genes, such as *Adamts4*, *Adamts5*, and *MMP13* (Fig. 5b). When simvastatin was embedded (SIM gel), competitive inhibition of HMGCR decreased the downstream enzyme *Mvk*, indicating downregulation of the cholesterol synthesis pathway, which is closely related to OA metabolism and affects joint health. We observed a slight upregulation in the expression of antioxidant genes in SIM gel. In addition, there was a significant upregulation of anabolic genes, such as *Acan* and *Col2a1*, confirming that the introduction of SIM gel into acetyl-CoA-stimulated primary chondrocytes resulted in a decrease in cholesterol metabolism (Fig. 5c). The introduction of SIM gel into N7KO and A12KO chondrocytes increased the expression of antioxidant genes, including *catalase* and *Sod2,* compared with the introduction of PD gel. Simultaneously, the expression levels of *Acan* and *Col2al* significantly increased with the introduction of SIM gel (Fig. 5d–e).

### 3.4 *In vivo* study of gel-sol transformation of SIM gel in OA mice

The *in vivo* studies were conducted to evaluate the potential performance of SIM gel by visualizing through naked-eye and delivering simvastatin for OA therapeutic applications. An *in vivo* biocompatibility test was initially performed to determine the possible inflammatory effects of SIM gel injection in mice. H&E staining and immunohistochemistry assays using cleaved caspase 3 and F4/80 showed no inflammation in the tissue after injection of SIM gel, which was similar to the tissue without SIM gel injection, confirming the biocompatibility of SIM gel (Fig. S14a). Furthermore, to assess systemic safety of SIM gel, we performed histological and immunohistochemical analyses of major organs 4 weeks after intra-articular injection. H&E staining of the liver, kidney, and spleen revealed preserved tissue architecture without signs of fibrosis, necrosis, or abnormal remodeling. F4/80 and cleaved caspase-3 immunostaining confirmed the absence of chronic macrophage infiltration and apoptotic cell death (Fig. S14b). These results demonstrate that SIM gel does not induce systemic inflammation or toxicity in distant organs following intra-articular administration. Although SIM gel contains MnO_2_ (a widely used biocompatible nanomaterials) that can be degraded into Mn^2+^ by CoASH in OA microenvironment, the generated Mn^2+^ (approximately 60 μg/mL) is still below a range shown to be tolerated (1.8–2.3 mg) and will be quickly excreted in the body without causing a long-term toxicity as reported by various works [[51], [52], [53], [54]]. Thus, SIM gel can be used safely for further OA theragnosis without altering joint microenvironment.

**Fig. 6:**
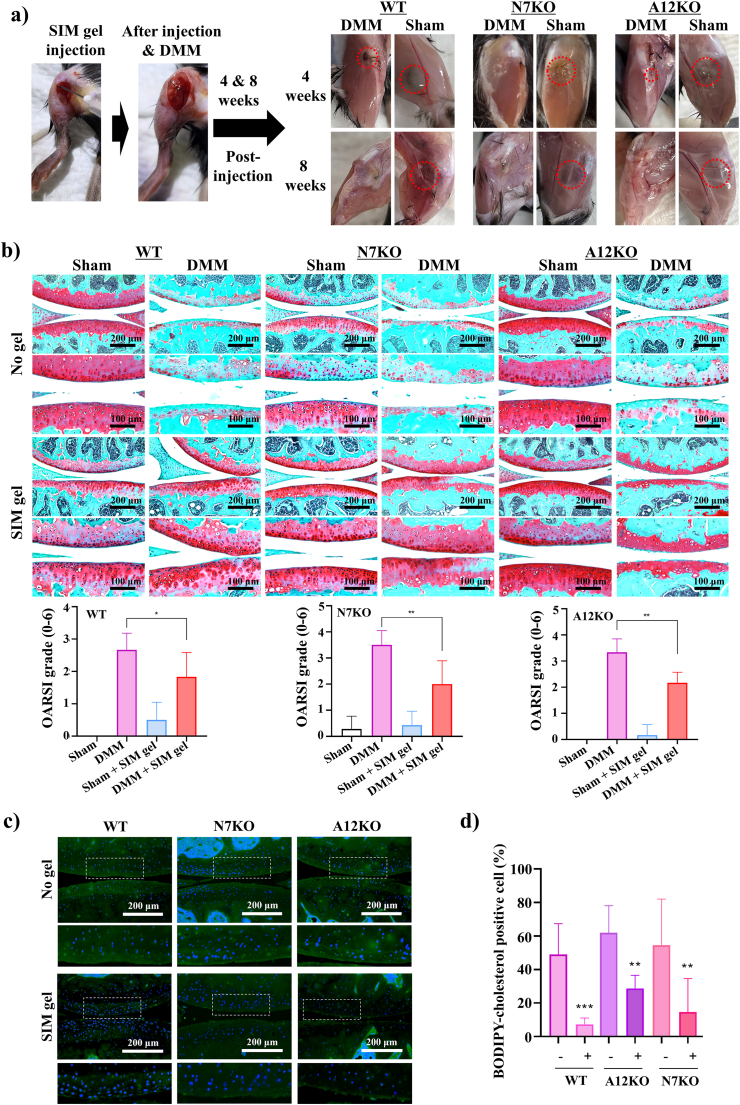
***In vivo* gel-sol transformation of SIM gel in OA mice cartilage. a)***In vivo* gel-sol transformation of SIM gel injected in DMM OA mice (WT, N7KO, and A12KO) at 4- and 8-week post-injection compared with sham mice (control). Red dashed circles indicate the visible gel region. **b)** Safranin O staining and OARSI score of DMM OA cartilage without and with injection of SIM gel. **c, d)** BODIPY-cholesterol staining assay of DMM OA cartilage without and with injection of SIM gel.

Next, *in vivo* validation was conducted using WT, N7KO, and A12KO OA mice models established by acetyl-CoA metabolism disruption *via* DMM surgery and compared with the control (Sham). The cartilage of each DMM and sham mice was monitored at 4 and 8 weeks post-SIM gel injection. As shown in Fig. 6a and S15a, the injected SIM gel started to lose its volume in the cartilage of DMM WT, N7KO, and A12KO mice at 4 weeks, with SIM gel in DMM N7KO completely disappearing. After 8 weeks, all SIM gels in each DMM mouse completely diminished as they changed to the sol form. These results contradict the findings in sham mice, in which SIM gel was stable and retained the gel form in the cartilage. The differences between sham and DMM mice were evident because of the abundant expression of CoASH or acetyl-CoA in DMM mice, triggering the gel-sol transformation of SIM gel. To evaluate the therapeutic superiority of our responsive delivery system, we compared SIM gel with free simvastatin in a DMM-induced OA model. As shown in Fig. S15b, joints treated with SIM gel retained significantly more proteoglycan content and showed better cartilage surface integrity than those treated with free simvastatin. These results highlight the importance of pathological microenvironment-triggered drug release and confirm the enhanced therapeutic efficacy of SIM gel compared to non-targeted delivery. Eight weeks postoperatively, the degree of cartilage degradation was assessed to determine the effects of SIM gel treatment (Fig. 6b, images). All DMM mice displayed a substantial loss of cartilage integrity and an increase in OA severity, as shown by the lower intensity of safranin O staining and increased OARSI scores, indicating severe cartilage matrix degradation and implications of acetyl-CoA metabolism abnormalities in OA progression. Conversely, all DMM mice exhibited markedly reduced cartilage degradation when injected with SIM gel despite OA induction. The SIM gel-injected groups showed more intensity of safranin O staining, suggesting the preservation of cartilage integrity. Furthermore, the OARSI score correlated with a significant decrease in cartilage degradation (Fig. 6b, graph). To validate the anabolic effects of SIM gel at the protein level, we performed immunofluorescence staining for COL2A1 and Acan in cartilage sections from OA joints (Fig. S16a). SIM gel-treated joints exhibited increased expression of both markers compared to untreated OA controls suggesting that SIM gel promotes chondrocyte anabolic activity and matrix restoration, consistent with the gene-level changes observed in our analysis. Consistent with the *in vitro* findings, *Sod2* expression, typically reduced in OA, was elevated following SIM gel administration into the cartilage of DMM mice (Fig. S16b–c). This suggests that statin gel mitigates cholesterol metabolism associated with acetyl-CoA dysregulation and exerts an antioxidative effect by upregulating *Sod2*. Moreover, the introduction of SIM gel significantly decreased BODIPY-cholesterol-positive cells in DMM-induced OA cartilage, demonstrating the effective therapeutic function of SIM gel in preserving the cartilage (Fig. 6c–d).

Based on these findings, the designed SIM gel can provide a unique approach on selectively controlling release of drug (simvastatin) based on the specific disease parameter (such as CoASH in OA) *via* distinct gel-sol transformation. Compared to the conventional drug-loaded temperature-sensitive injectable hydrogels, which rely on the swelling-controlled mechanisms and suffer from uncontrolled drug release in OA microenvironment owing to their structural characteristics, SIM gel offers on-demand controlled release of simvastatin triggered by specific level of CoASH that is closely correlated with the severity of OA (can be burst or sustain releases). Hence, this approach effectively lowers cholesterol amount which influences the acetyl-CoA-related metabolic pathways for the treatment of OA. Also, unlike another temperature-sensitive injectable hydrogels, the distinct gel-sol transition in OA microenvironment at physiological temperature can be used as a naked-eye indication for *in situ* monitoring of OA without any additional instrumentation, complementing the function of SIM gel as an OA theragnostic system. For supporting future development and addressing current limitations of this system, further studies including more comprehensive *in vivo* and clinical studies should be conducted to specifically evaluate the theragnostic performance of SIM gel in more complex OA patient or OA-bearing model conditions, such as those who possess disease complication which possibly alters the OA microenvironment and pathogenesis. Also, in the case of the possibility to combine this system with diagnostic devices (such as wireless electrochemical system) for *in situ* monitoring, we need to improve the conductivity of the nanoparticle in SIM gel to obtain high sensitivity, as this current material possessed quite low conductivity which resulted in the low detection sensitivity and low signal transmission in wireless electrochemical system.

## 4 Conclusions

We have successfully designed a naked-eye diagnostic and controlled therapeutic approach for OA using an injectable SIM gel by manipulating a deregulated lipid metabolism-induced gel-to-sol phase transition in OA cartilage at body temperature (37 °C). SIM gel, which was distinct from conventional temperature-sensitive injectable hydrogel such as HGC gel, showed a gel-sol transformation at 37 °C triggered by the high expression of CoASH in OA chondrocytes, which stimulated the cleavage of MnO_2_ into Mn^2+^ in SIM gel and affected the hydrophobicity–hydrophilicity of SIM gel. This physical change can be visualized for the diagnosis of OA, as proven by the sol formation of SIM gel after incubation in OA chondrocytes, including WT-induced acetyl-CoA (WT + CoA), N7KO, and A12KO. *In vitro* gene expression studies revealed that SIM gel is capable of downregulating the transcriptional levels of antioxidant enzymes (*catalase* and *Sod2*), cartilage degradation genes (*Adamts4*, *Adamts5*, and *MMP13*), and cholesterol synthesis-related enzymes (*Mvk*), which are closely related to cartilage degradation. *In vivo* experiments further demonstrated the gel-sol transformation in DMM mice (WT, N7KO, and A12KO mice), indicated by the disappearance of SIM gel at 4 and 8 weeks after injection into the cartilage, which was contrary to the sham mice (control) that retained the stable gel form. Furthermore, reduced cartilage degradation, *Sod2* expression, and BODIPY-cholesterol-positive cells were observed in DMM mice despite the OA induction, confirming the capability of SIM gel to cure OA cartilage by releasing simvastatin from SIM gel. Thus, the as-synthesized SIM gel provides a new approach for developing a synergistic diagnosis and treatment of OA, which has the potential to be applied for joint dysfunction theragnostic.

Ethics approval and consent to participate

All animal studies followed the approval from the Wonkwang University Animal Care and Use Committee and the institutional guidelines (WKU23-37).

## CRediT authorship contribution statement

**Akhmad Irhas Robby:** Writing – review & editing, Writing – original draft, Validation, Methodology, Investigation, Funding acquisition, Conceptualization. **Ee Hyun Kim:** Validation, Methodology, Investigation, Conceptualization. **Kang Moo Huh:** Writing – review & editing, Methodology, Conceptualization. **Eun-Jung Jin:** Writing – review & editing, Writing – original draft, Supervision, Resources, Methodology, Data curation, Conceptualization. **Ki Dong Park:** Validation, Supervision, Methodology, Formal analysis. **Sung Young Park:** Writing – review & editing, Writing – original draft, Supervision, Resources, Methodology, Funding acquisition, Data curation, Conceptualization.

## Declaration of competing interest

The authors declare that they have no known competing financial interests or personal relationships that could have appeared to influence the work reported in this paper.
