# Supplementary material for: Mevalonate pathway-triggered phase transition of injectable hydrogel for cholesterol-downregulated therapy of osteoarthritis
Source: Bioact Mater. 2025 Jun 28;51:876–88. doi: 10.1016/j.bioactmat.2025.06.047 (PMC12269283; doi:10.1016/j.bioactmat.2025.06.047)
Supplement: Multimedia component 1 [file mmc1.docx]

**Supporting Information**

**Mevalonate Pathway-triggered Phase Transition of Injectable Hydrogel for Cholesterol-downregulated Therapy of Osteoarthritis**

**Experimental Section**

*Quantitative real-time PCR (qRT-PCR) analysis*

cDNA was synthesized with total RNA extracted from chondrocytes and qRT-PCR was performed on ABI StepOnePlus instrument (Applied Biosystems). The primers used were as follows: *Acan*, Forward:5’-GAGACTTCTGCCTCTGGAATAG-3’, Reverse:5’-CTCCAGAAGGAATCCCACTAAC-3’, *Adamts4*, Forward:5’-GCATTCCATGGTACAGGGTTA-3’, Reverse:5’-AGTTGACAGGGTTTCGGATG-3’, *Adamts5*, Forward:5’-TGCCACAGACCCAACTAAAG-3’, Reverse:5’-CCATGGCTGATGACAGAGTT-3’, *Catalase*, Forward:5’-GATGGTAACTGGGATCTTGTGG-3’, Reverse:5’-GTGGGTTTCTCTTCTGGCTATG-3’, *Col2a1*, Forward:5’-CTGGTTTGGAGAGACCATGAA-3’, Reverse:5’-GAGGAAAGTCATCTGGACGTTAG-3’, *Comp*, Forward:5’-CGTGGGCTGGAAGGATAAA-3’, Reverse:5’-TACTAGCTCAGGACCCTCATAG-3’, *Hmgcr*, Forward:5’-CTTGTGGAATGCCTTGTGATTG-3’, Reverse:5’-AGCCGAAGCAGCACATGAT-3’, *Hmgcs*, Forward:5’-AAATGCCAGACCTACAGGTGG-3’, Reverse:5’-ATGCTGCATGTGTGTCCCA-3’, *Mmp13*, Forward:5’-CCCTGATGTTTCCCATCTATACC-3’, Reverse:5’-TTCATCGCCTGGACCATAAAG-3’, *Mvk*, Forward:5’-GGAGCAACTGGAGAAGCTAAA-3’, Reverse:5’-TGCCAGGTACAGGTAGAGAA-3’, *Rn18s*, Forward:5’-CCAGTAAGTGCGGGTCATAAG-3’, Reverse:5’-GGCCTCACTAAACCATCCAA-3’, *Sod2*, Forward:5’-AGCGTGACTTTGGGTCTTT-3’, Reverse:5’-AGCGACCTTGCTCCTTATTG-3’.


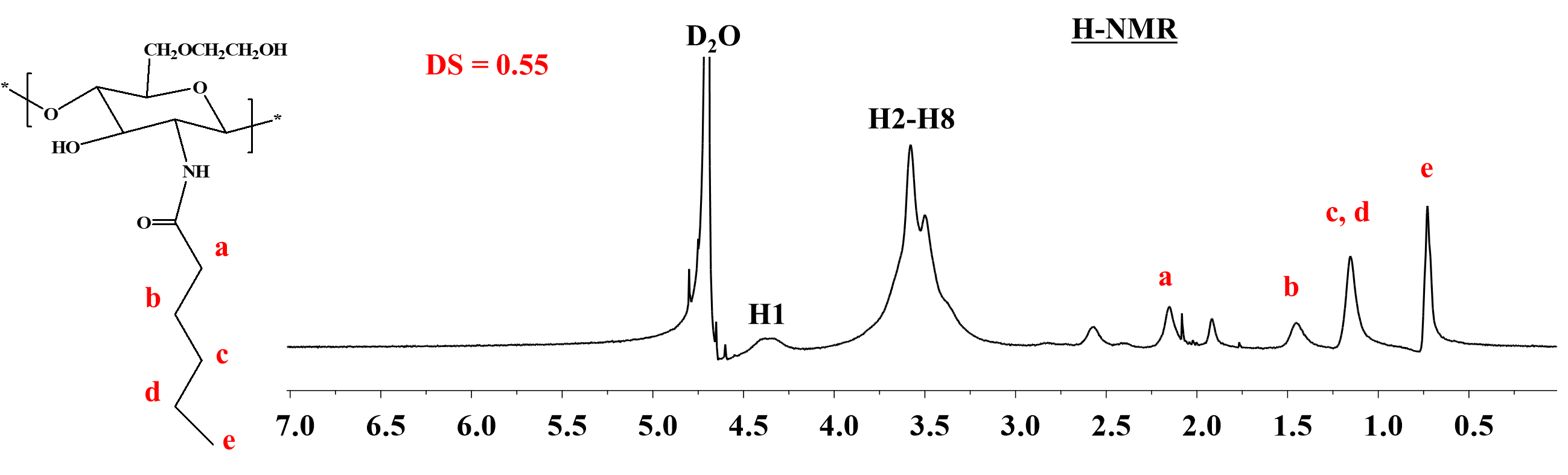


**Figure S1.** ^1^H-NMR spectra of as-synthesized HGC.


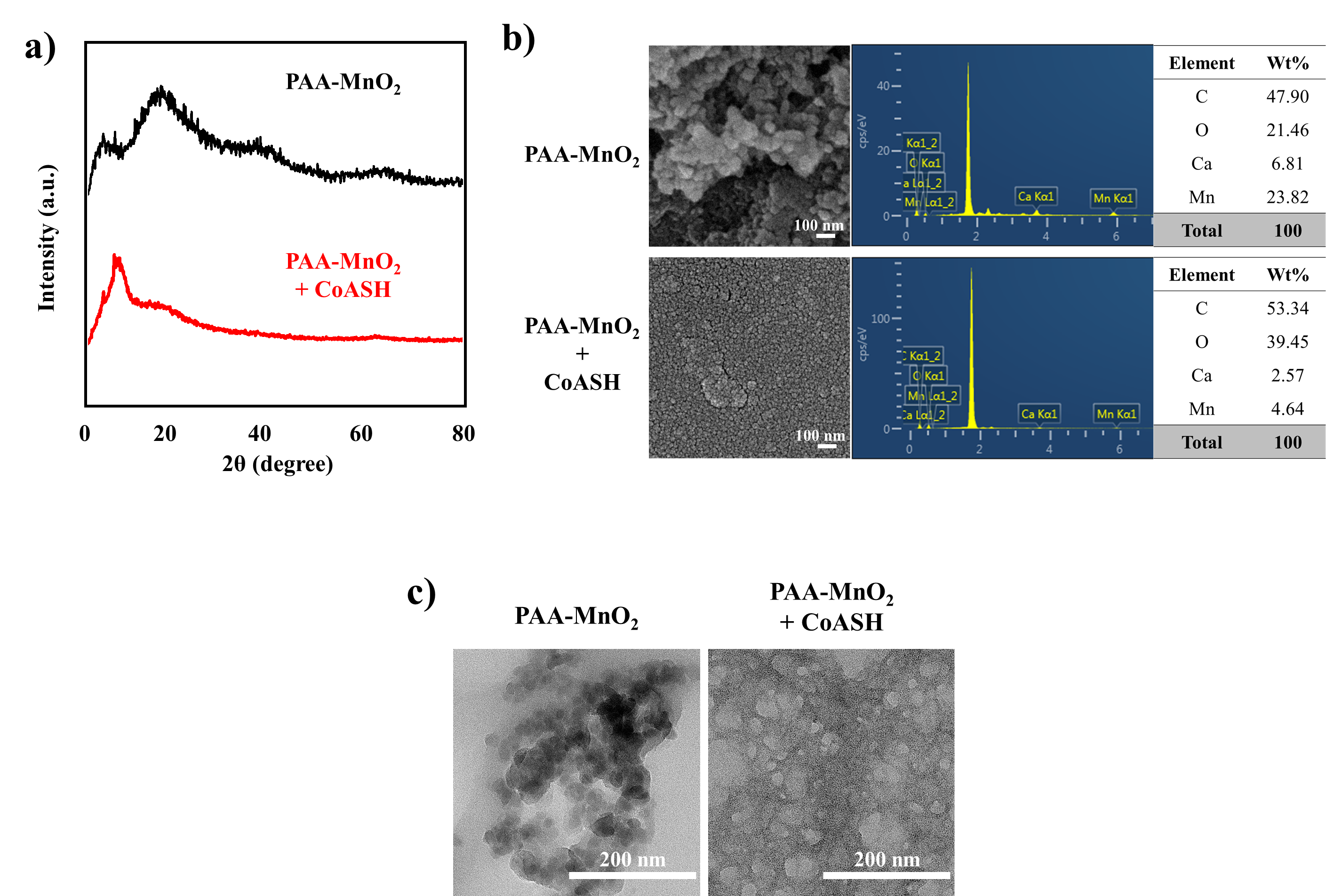


**Figure S2.** **a)** XRD spectra, **b)** SEM-EDX profiles, and **c)** TEM images of PAA-MnO_2_ nanoparticles before and after treatment with CoASH (10 mM).

**Figure S3.** Simvastatin release profile from SIM@PAA-MnO_2_ nanoparticles after treatment with CoASH (1, 5, 10 mM).

**Figure S4.** Swelling ratio of SIM gel.


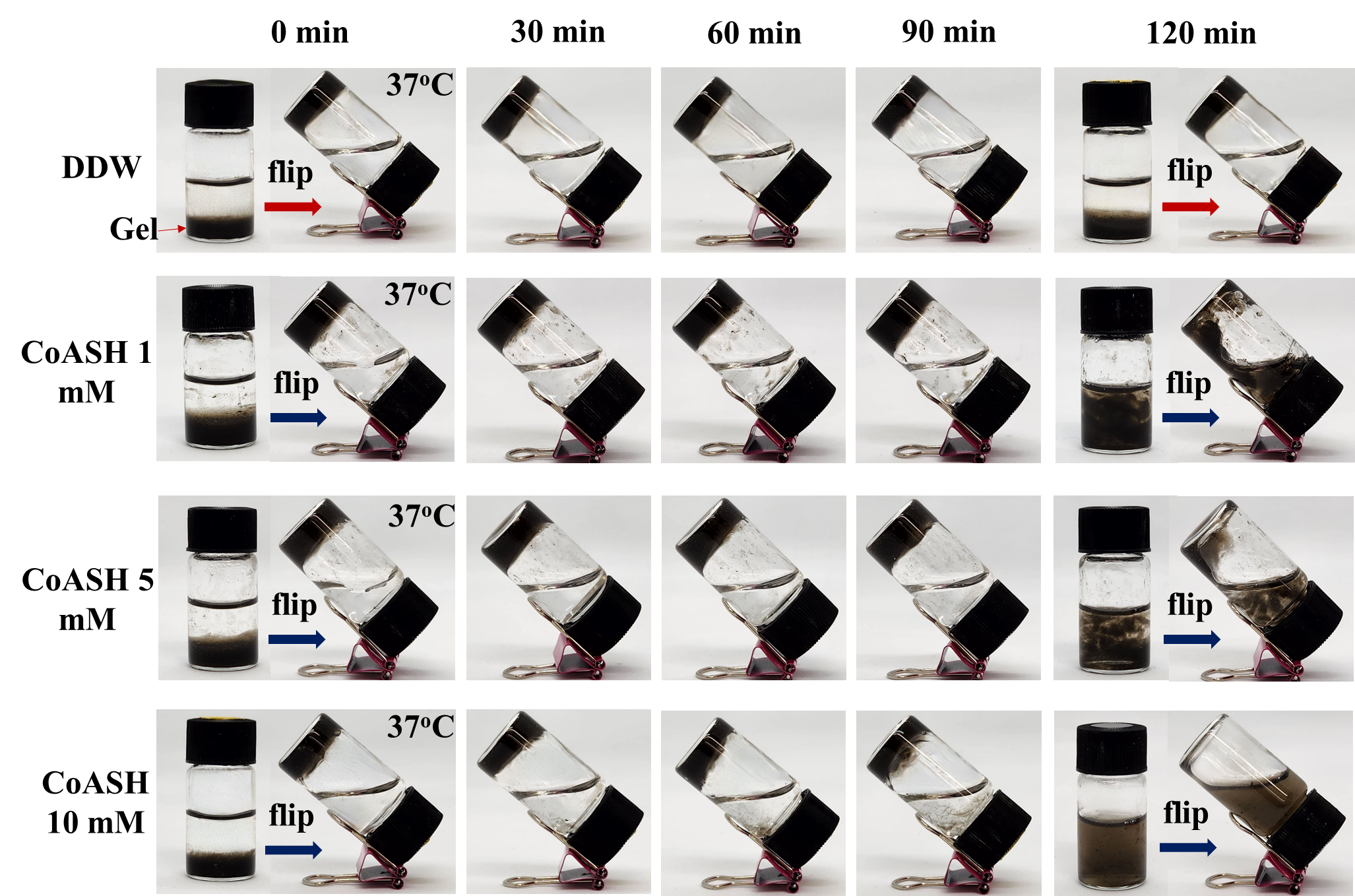


**Figure S5.** Stability of SIM gel in various concentrations of CoASH and incubation times at 37^o^C.


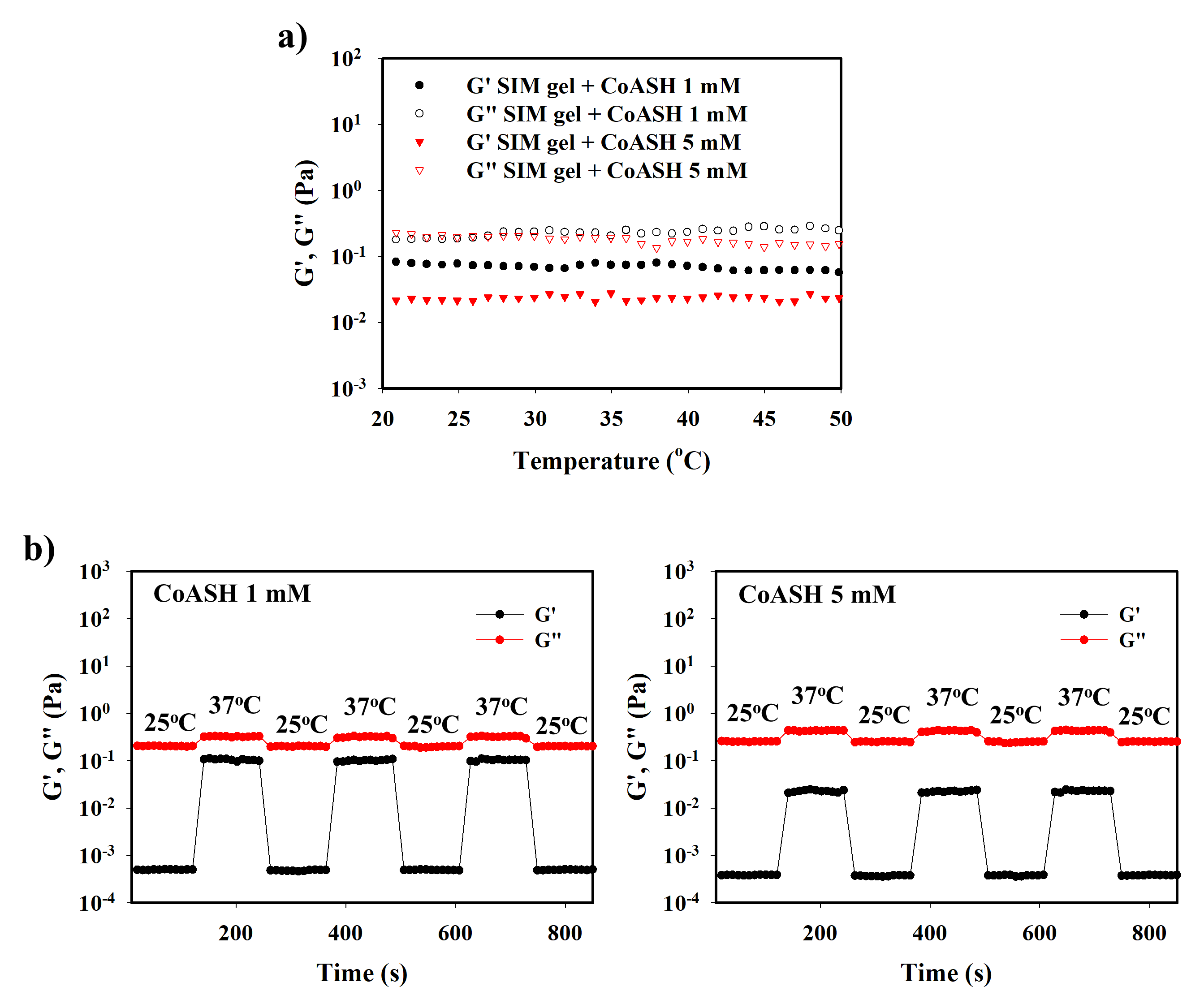


**Figure S6.** **a)** Rheological temperature sweep at 37^o^C, and **b)** Rheological reversibility test (25^o^-37^o^C) of SIM gel treated with CoASH 1 and 5 mM for 2 h.


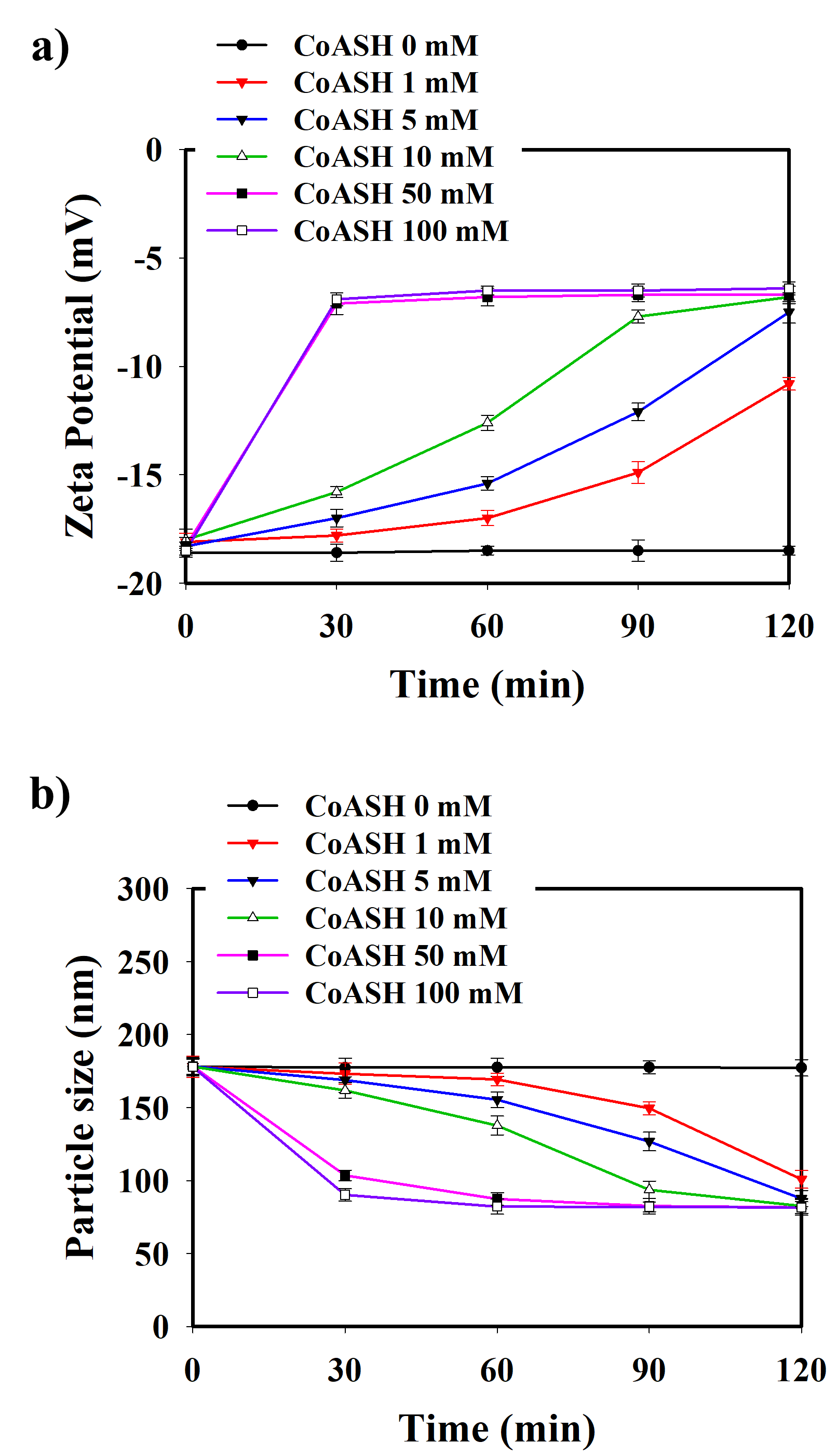


**Figure S7.** **a)** Zeta potential and **b)** DLS measurements of SIM gel (0.001 mg/mL) treated with various concentrations of CoASH for 2 h at 37^o^C.


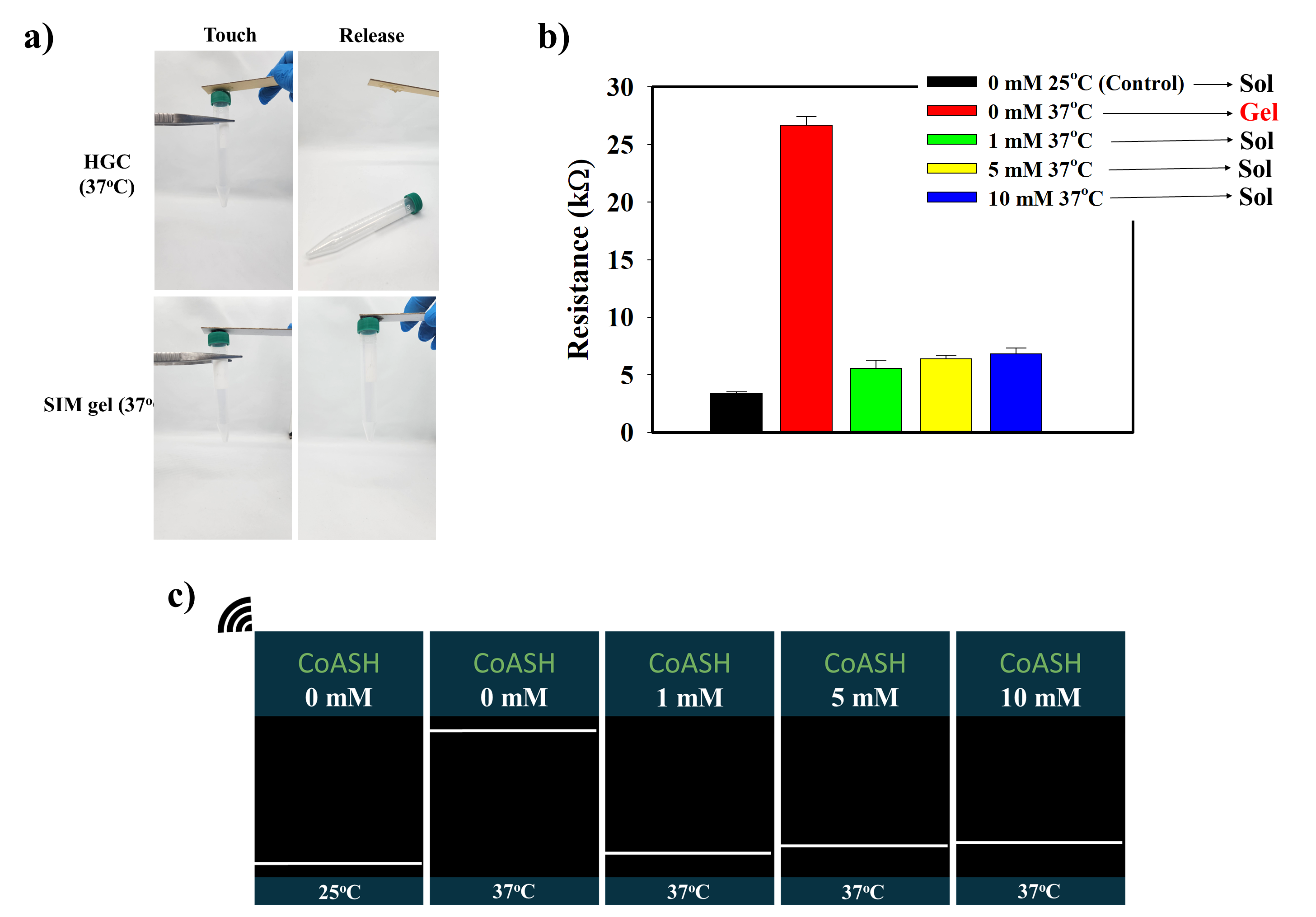


**Figure S8.** **a)** Adhesive test of SIM gel compared to HGC gel at 37^o^C. **b)** Sourcemeter measurement and **c)** wireless sensing (showed as resistance graph) of SIM gel treated with CoASH 1, 5 and 10 mM for 2 h at 37^o^C.


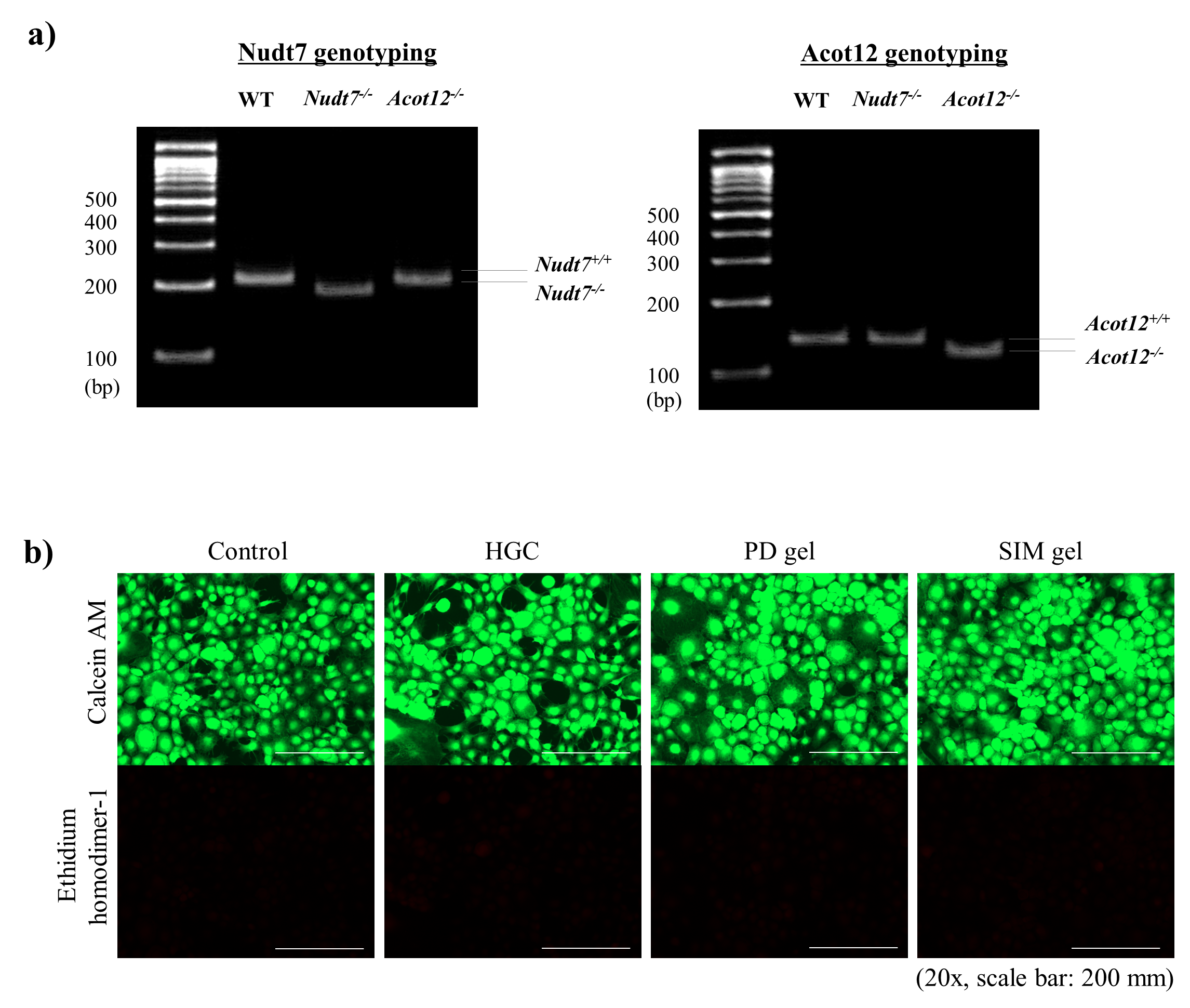


**Figure S9.** **a)** Genotyping analysis of Nudt7 (N7KO) and Acot12 (A12KO) knockout mice. **b)** *In vitro* live and dead assay of articular chondrocytes treated with HGC, PD gel and SIM gel.


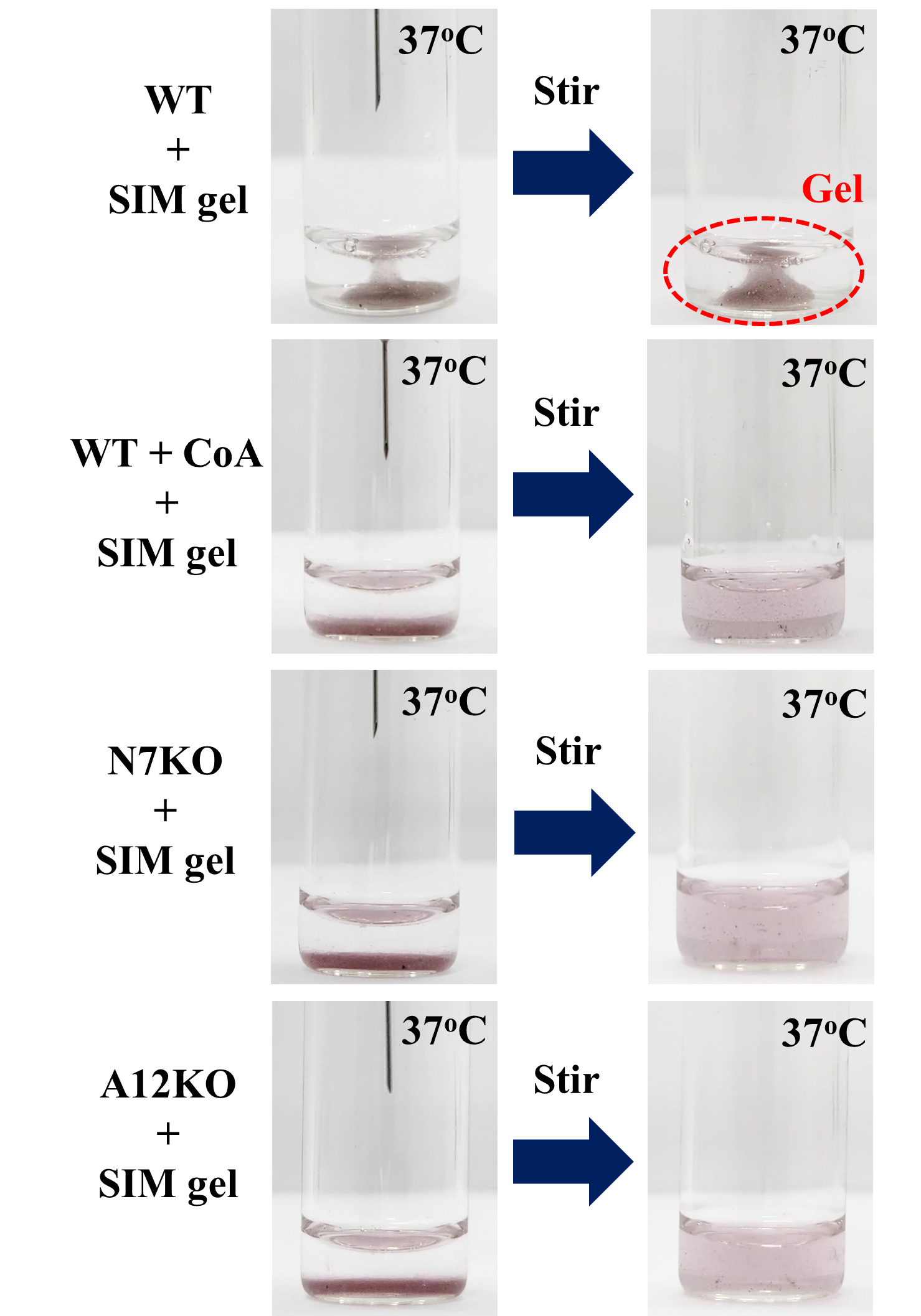


**Figure S10.** *In vitro* gel-sol transformation of SIM gel treated with OA chondrocytes model (WT, WT + CoA, N7KO and A12KO) for 24 h at 37^o^C assessed with a dissolution test.


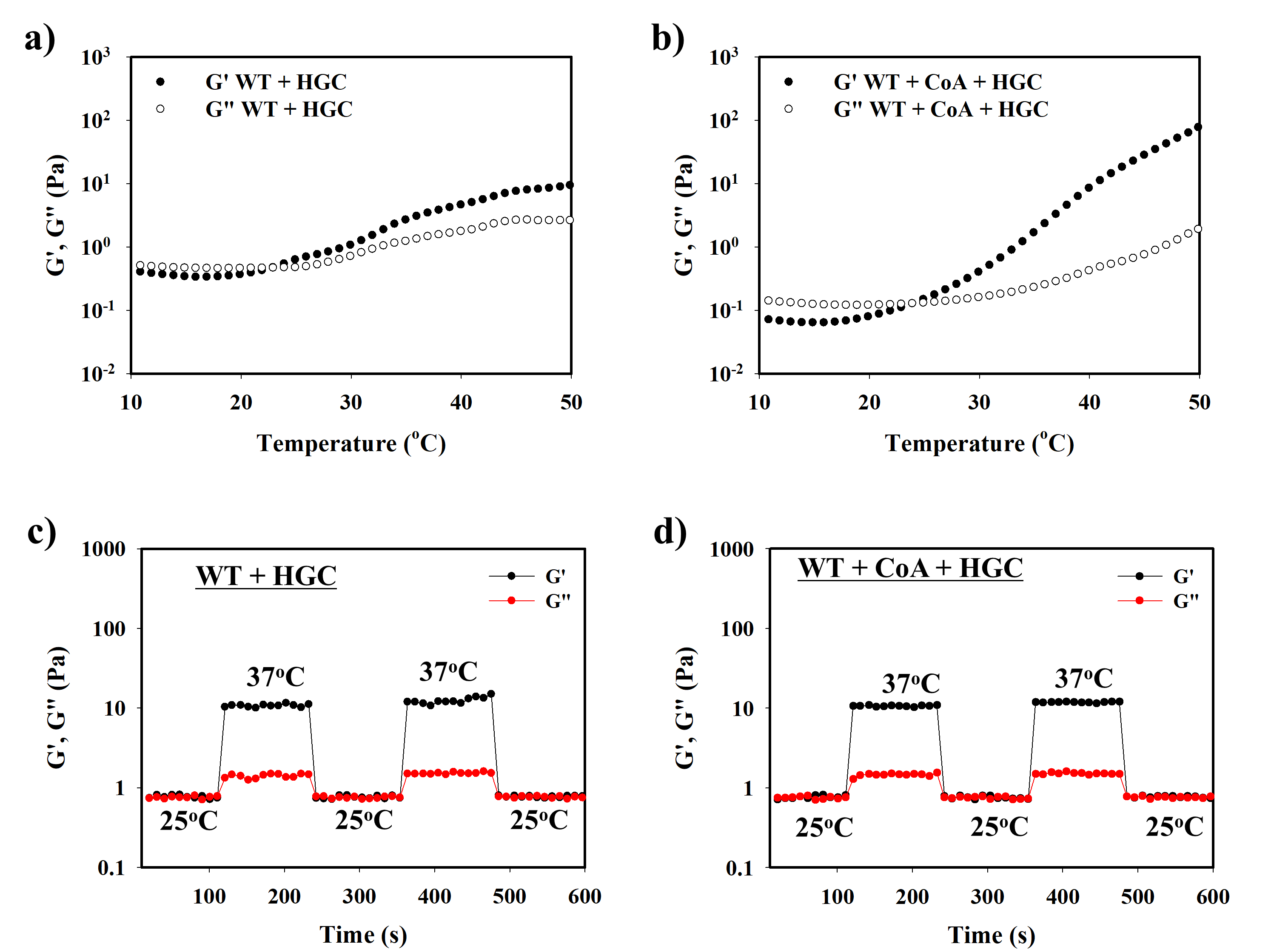


**Figure S11.** *In vitro* temperature sweep of **a)** WT and **b)** WT + CoA-treated HGC gel. In vitro reversibility test of **c)** WT and **d)** WT + CoA-treated HGC gel.


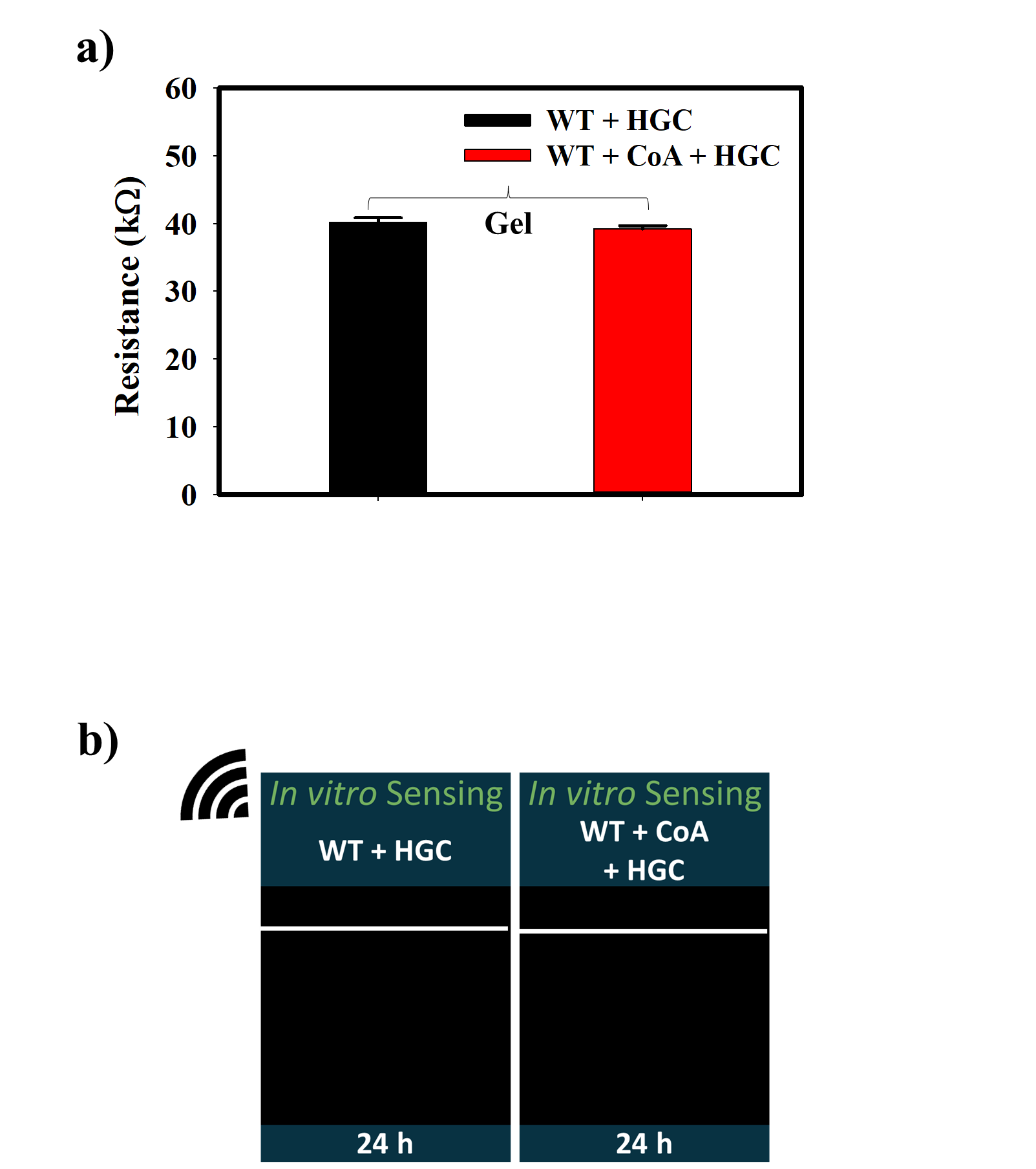


**Figure S12.** **a)** Sourcemeter measurement, and **b)** wireless sensing (showed as resistance graph) of HGC gel treated with WT and WT + CoA chondrocytes (24 h, 37^o^C).


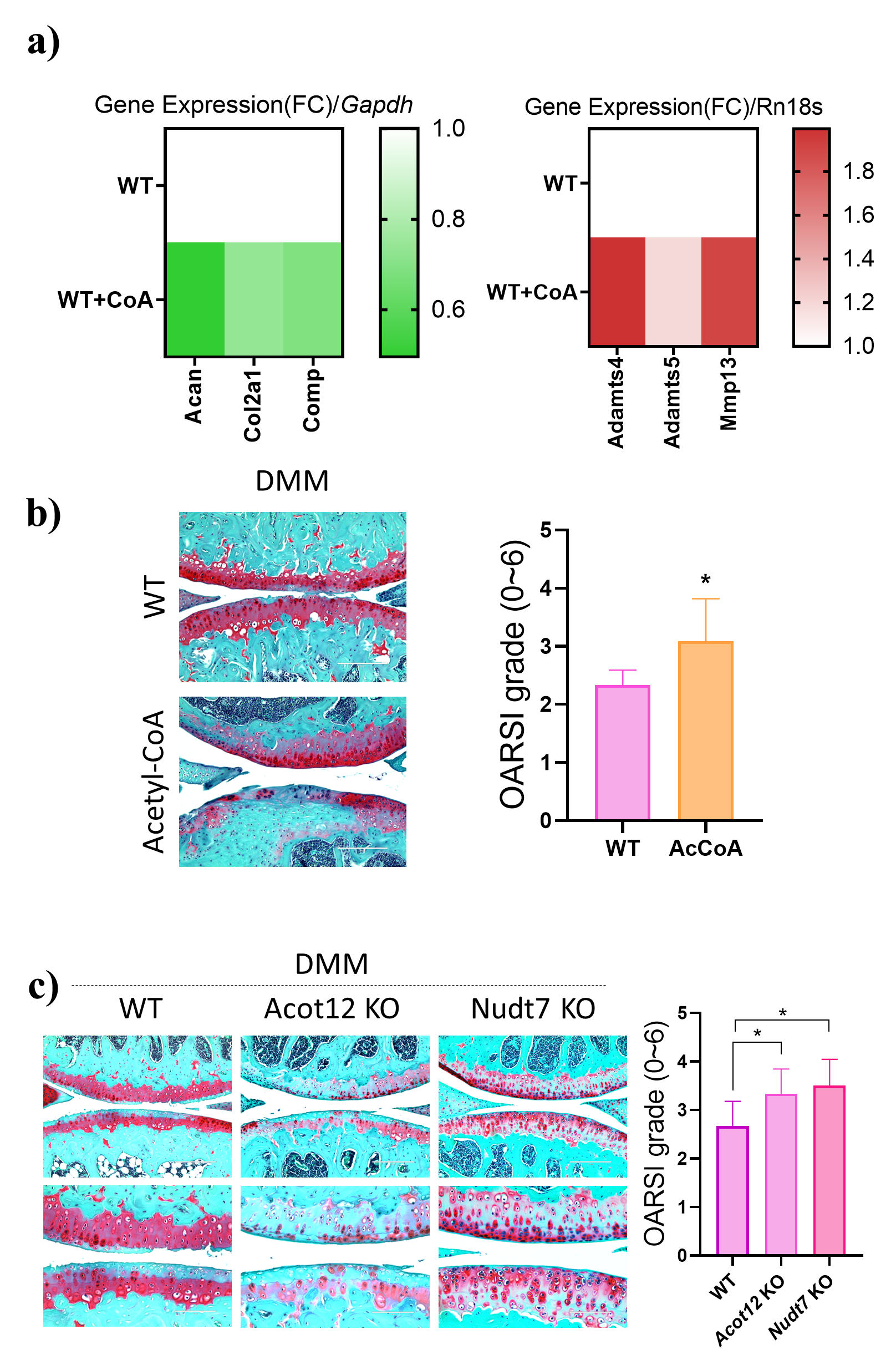


**Figure S13.** **a)** transcriptional gene expression of WT and WT + CoA OA cartilage. Safranin O staining and OARSI scoring of **b)** DMM WT and WT-CoA cartilages, and **c)** DMM WT, N7KO and A12KO cartilage.


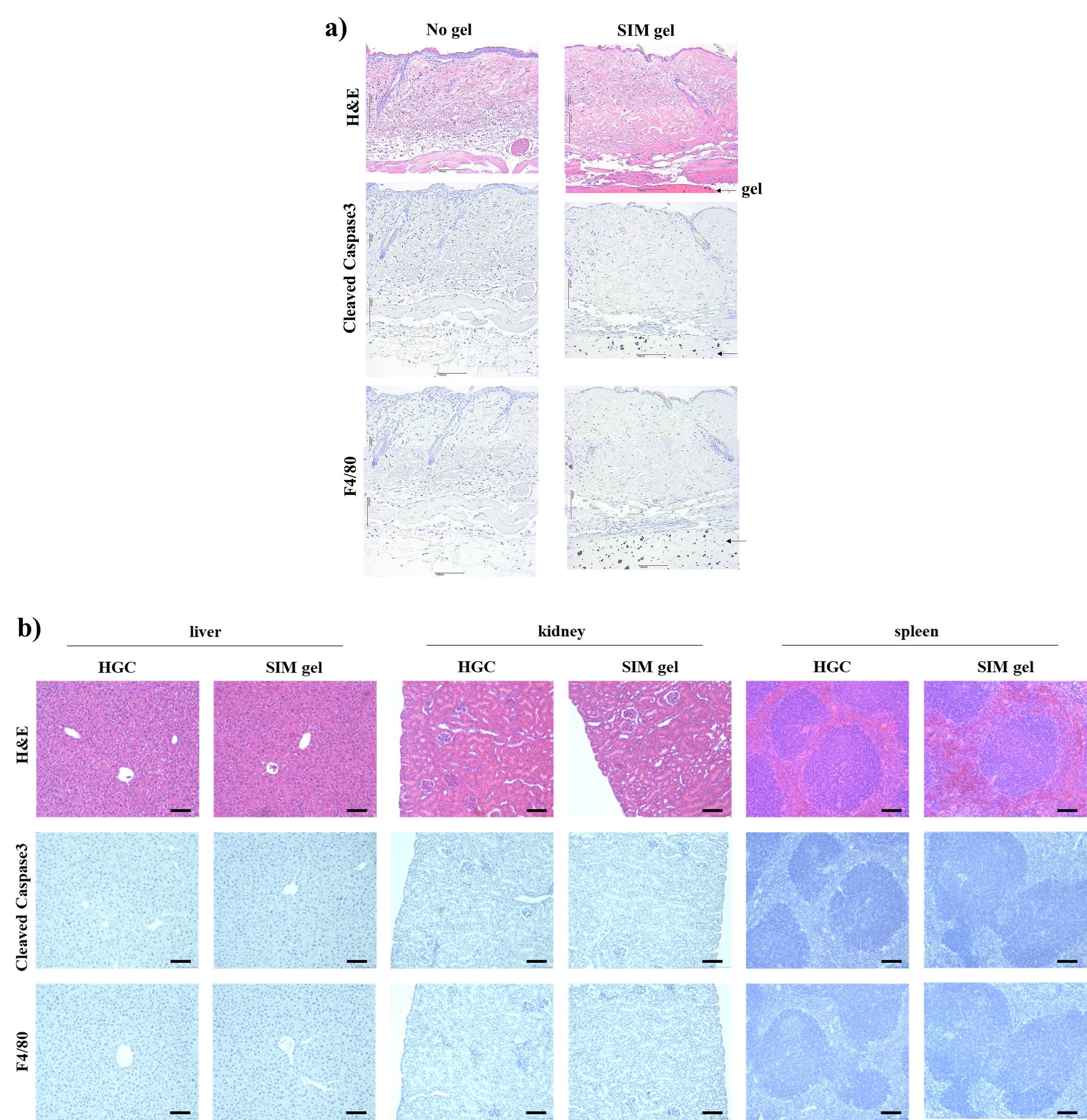


**Figure S14.** Histological and immunohistochemical evaluation of systemic biocompatibility following intra-articular injection of SIM gel: **a)** *In vivo* biocompatibility assay of joint tissue without and with SIM gel injection. **b)** Representative Hematoxylin and eosin (H&E) staining, F4/80 immunostaining, and Cleaved caspase-3 immunostaining of the liver, kidney, and spleen 4 weeks after injection of SIM gel. Scale bars: 100 µm. Images are representative of n = 3 mice per group.


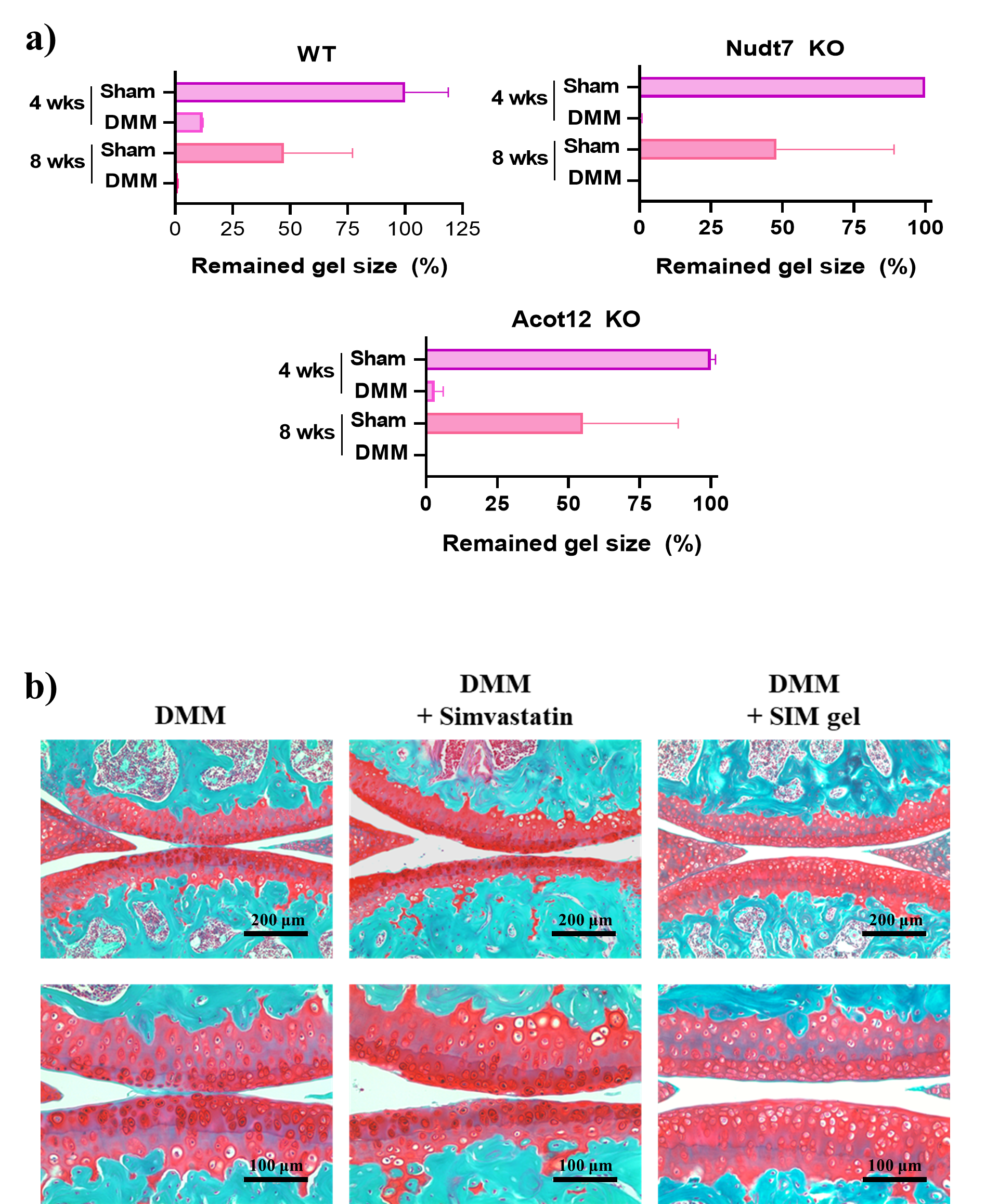


**Figure S15.** *In vivo* **a)** change in volume of SIM gel, and **b)** Safranin-O/Fast Green staining of articular cartilage in DMM-operated mice treated with free simvastatin and SIM gel at 4 weeks post-injection.


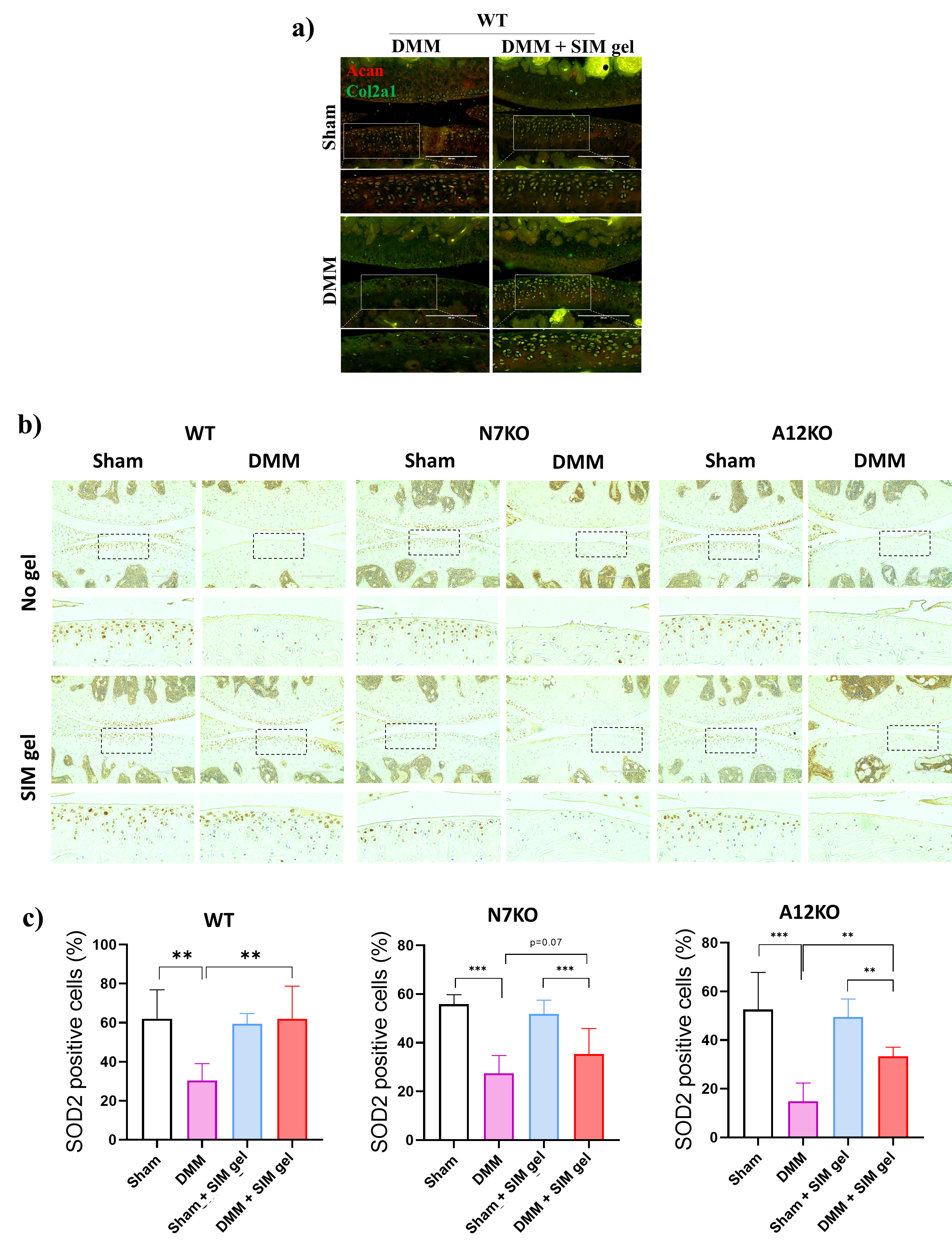


**Figure S16.** *In vivo* **a)** immunofluorescence of Acan (TRITC) and Col2a1 (FITC) in WT DMM cartilage, **b)** Sod2 assay and **c)** quantification of Sod2 positive cell in WT, N7KO and A12KO DMM cartilage.
